# Dynamism of Metabolic Carbon Flow of Starch and Lipids in *Chlamydomonas debaryana*

**DOI:** 10.3389/fpls.2021.646498

**Published:** 2021-03-30

**Authors:** Naoki Sato, Masakazu Toyoshima

**Affiliations:** Department of Life Sciences, Graduate School of Arts and Science, University of Tokyo, Meguro, Japan

**Keywords:** biofuel production, green alga, isotopomer analysis, lipid biosynthesis, metabolic analysis, stable isotope

## Abstract

Microalgae have the potential to recycle CO_2_ as starch and triacylglycerol (TAG), which provide alternative source of biofuel and high added-value chemicals. Starch accumulates in the chloroplast, whereas TAG accumulates in the cytoplasmic lipid droplets (LD). Preferential accumulation of starch or TAG may be achieved by switching intracellular metabolic carbon flow, but our knowledge on this control remains limited. Are these two products mutually exclusive? Or, does starch act as a precursor to TAG synthesis, or vice versa? To answer these questions, we analyzed carbon flow in starch and lipids using a stable isotope ^13^C in *Chlamydomonas debaryana* NIES-2212, which accumulates, without nutrient limitation, starch in the exponential growth phase and TAG in the stationary phase. Pulse labeling experiments as well as pulse labeling and chase experiments were conducted, and then, gas chromatography-mass spectrometry (GC-MS) analysis was performed on starch-derived glucose and lipid-bound fatty acids. We exploited the previously developed method of isotopomer analysis to estimate the proportion of various pools with different isotopic abundance. Starch turned over rapidly to provide carbon for the synthesis of fatty acids in the exponential phase cells. Most fatty acids showed rapid and slow components of metabolism, whereas oleic acid decayed according to a single exponential curve. Highly labeled population of fatty acids that accumulated during the initial labeling decreased rapidly, and replaced by low abundance population during the chase time, indicating that highly labeled fatty acids were degraded and the resulting carbons were re-used in the re-synthesis with about 9-fold unlabeled, newly fixed carbons. Elongation of C16–C18 acids *in vivo* was indicated by partially labeled C18 acids. The accumulation of TAG in the stationary growth phase was accounted for by both *de novo* synthesis and remodeling of membrane lipids. These results suggest that *de novo* synthesis of starch and TAG was rapid and transient, and also almost independent to each other, but there is a pool of starch quickly turning over for the synthesis of fatty acids. Fatty acids were also subject to re-synthesis. Evidence was also provided for remodeling of lipids, namely, re-use of acyl groups in polar lipids for TAG synthesis.

## Introduction

Photosynthetic organisms, such as plants, algae, cyanobacteria and anoxygenic bacteria, recycle CO_2_ under the sunlight to provide organic matters for the biosphere and human life. Currently, industrial production of biofuels such as bioethanol and biodiesel from the microalgal products, oil and starch, are extensively studied (Siaut et al., 2011; Aikawa et al., 2013; Bhowmick et al., 2016; Sato et al., 2017). Plant and microalgal oil is mainly triacylglycerol (TAG) consisting of long chain fatty acids. The overall picture of the complex metabolic pathway leading to TAG synthesis was elucidated in plants (Lung and Weselake, 2006; Li-Beisson et al., 2013; Bates, 2016), but this might not be simply adapted to microalgae, because microalgae are unicellular organisms that accumulate both starch and TAG within a single cell, whereas the sink and source organs are separated in plants. The metabolic pathways in microalgae have to be established using information of comparative genomics that links orthologs in algae, bacteria, protists and plants (Riekhof et al., 2005; Sato and Moriyama, 2007; Misra et al., 2012; Sato and Obayashi, 2021).

In plants, TAG is synthesized by two major pathways (Li-Beisson et al., 2013): namely, the acylation of glycerol-3-phosphate with acyl-CoA in the *de novo* pathway called Kennedy pathway, and the remodeling pathway in which phosphatidylcholine (PC) acts as an intermediate that provides fatty acids and/or diacylglycerol (DAG) for the synthesis of TAG (Bates et al., 2013). In plants, TAG is typically accumulated as lipid droplets (LD) in the cytosol of developing seeds, although LD is supposed to emerge by budding from the endoplasmic reticulum (ER). LD is typically a spherical globule of 0.1–1 μm in diameter, mainly consisting of TAG and surrounded by a half unit membrane containing oleosin and other specific proteins (Chapman et al., 2012). LD is also present in the cytosol of microalgae (Moriyama et al., 2018), but the proteins in the LD membrane are not related to oleosin. A major LD protein is called MLDP in *Chlamydomonas reinhardtii* (Moellering and Benning, 2010). TAG is also known to be present as a minor component of plastoglobules, which are small lipid droplets present in chloroplasts, consisting of plastoquinone, tocopherol, and phytyl esters (Bréhélin et al., 2007; Lohscheider and Bártulos, 2016).

*Chlamydomonas reinhardtii* has been used as a model organism for the production of TAG (Li et al., 2012; Merchant et al., 2012; Sakurai et al., 2014; Li-Beisson et al., 2015; Juergens et al., 2016) and starch (Van den Koornhuyse et al., 1996; Hicks et al., 2001) under nutrient-limited conditions such as nitrogen deficiency, which is also known to increase accumulation of TAG in various algae (Tornabene et al., 1983; Yusuf, 2007; Hu et al., 2008; Aikawa et al., 2012; Msanne et al., 2012; Iwai et al., 2014). In these cases, membrane lipids are remodeled to form TAG under limitation of *de novo* synthesis of lipids. Some algae are known to accumulate TAG or other lipid materials without severe limitation of nutrients. *Nannochloropsis* sp., *Botryococcus braunii*, *Dunaliella primolecta*, and *Nitzschia* sp. are representatives of many oil-accumulating microalgae known to date (for a review, see Menetrez, 2012). Different strains of *Chlamydomonas debaryana* also accumulate large amount of TAG in the stationary growth phase, but this is not caused by nutrient deficiency (Zhang et al., 2014; Toyoshima and Sato, 2015). In this respect, *C. debaryana* is different from *C. reinhardtii*, which does not accumulate TAG in the stationary growth phase without nutrient limitation (Sakurai et al., 2014). A recent research using gel encapsulation showed that increased cell density promoted cell growth and TAG accumulation in *C. debaryana* but not *C. reinhardtii* (Yoshitomi et al., 2020). In another study (Toyoshima and Sato, 2018), we tried to optimize TAG production in this alga, and found that initial starch accumulation was followed by TAG accumulation that accompanied degradation of starch. We suspected a close relationship between starch metabolism and TAG synthesis. However, no clear correlation was detected between maximum levels of starch and TAG accumulation under nitrogen limitation in various strains and mutants of *C. reinhardtii* (Siaut et al., 2011). This necessitates labeling studies on the carbon flow in starch and TAG. An NMR study using [^13^C] acetate and bicarbonate was reported in *C. reinhardtii* and suggested the importance of autotrophic nutrition in starch and TAG accumulation (Singh et al., 2014). In this context, we found *C. debaryana* a good model system to study biosynthesis and carbon flow in starch and TAG under photoautotrophic condition without nutrient limitation.

Labeling study using ^13^C is potentially useful and powerful. Mass spectral analysis of incorporated ^13^C in various different parts of molecules will provide detailed information on the carbon flow in labeling-and-chase experiments. We developed a new method of ^13^C isotopomer analysis, which is implemented in the C13dist program (Sato et al., 2016), focusing on the isotopic abundance (*p*), to trace differently labeled carbon pools in the cell. An isotopomer is a molecule containing a defined number of ^13^C. A single value of *p* defines a binomial distribution of isotopomers. The C13dist program is intended to estimate the distribution of *p* from the observed isotopomer distribution. Carbon pools with different *p* values can be analyzed as separate pools. In this respect, a single isotope ^13^C can be used as multiple tracers. In the present study, we exploited this ^13^C isotopomer analysis to study synthesis of starch and lipids, potential conversion of starch to lipids, as well as remodeling of membrane lipids to TAG.

## Materials and Methods

### Growth of Organism

Cells of *C. debaryana* NIES-2212 (Yumoto et al., 2013) were grown under continuous light (about 50 μmol m^−2^ s^−1^) in the Modified Bristol’s Medium (MBM; Watanabe, 1960) under aeration with 1.0% (v/v) CO_2_ at 25°C. The actual constituents of the MBM that we use were described previously (Sakurai et al., 2014).

### Photosynthetic Labeling With Sodium [^13^C]bicarbonate

**Figure 1:**
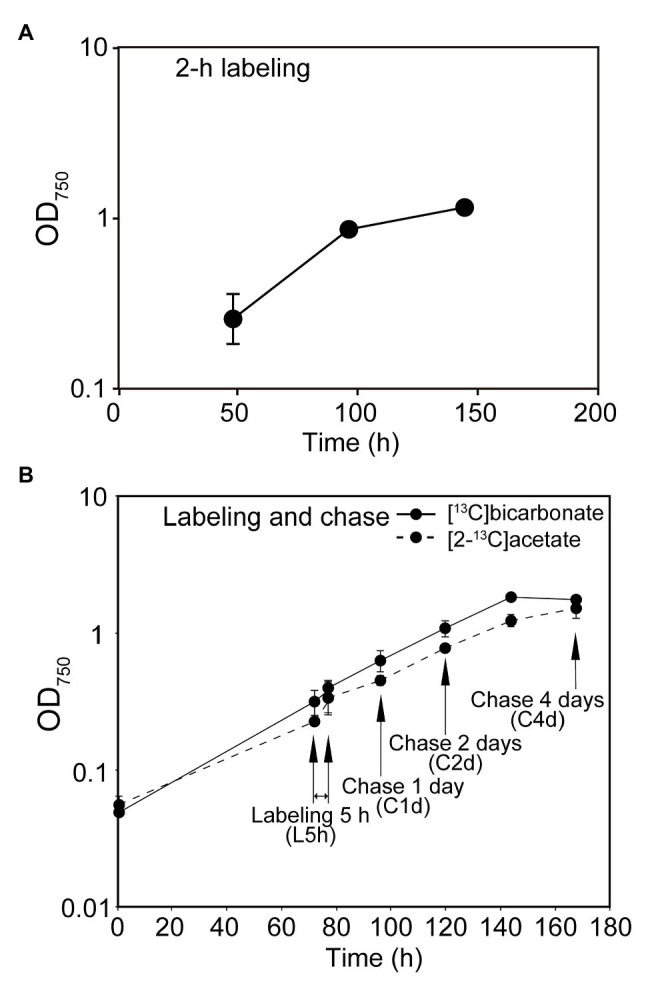
Growth of cells used in the experiments. Turbidity at 750 nm is shown at each sampling point. **(A)** Labeling for 2 h. **(B)** Labeling for 5 h and chase.

The cells (200-ml culture) grown under the photoautotrophic conditions at 48, 96, and 144 h after inoculation (Figure 1A) were collected by centrifugation (600 × *g*, 5 min, 4°C) and then resuspended in 200 ml of fresh medium containing 13 mM sodium [^13^C]bicarbonate (99% ^13^C, Cambridge Isotope Laboratories, Inc., Tewksbury, MA, United States). The cells were allowed to grow for 2 h in the light with gentle stirring in a tightly closed flask. Cells were harvested by centrifugation. Total lipids were extracted according to the method of Bligh and Dyer (1959) as described in Toyoshima and Sato (2015). After partitioning, the interface fluff containing proteins, starch and other insoluble materials was recovered as a starch source, and stored at −80°C until analysis. The chloroform phase (lipids) was evaporated under reduced pressure. The lipids were dissolved in 0.2 ml of chloroform/methanol (2:1, by volume) plus 0.2 ml of ethanol, and stored at −20°C until use. The experiments were performed in triplicates.

### Labeling and Chase Experiments With [^13^C]bicarbonate and [2-^13^C]acetate

The cells (300-ml culture) grown under the photoautotrophic conditions for 72 h after inoculation (Figure 1B) were collected by centrifugation (600 × *g*, 5 min, 4°C) and then resuspended in 300 ml of fresh medium containing 20 mM sodium [^13^C]bicarbonate or sodium [2-^13^C]acetate (99% ^13^C, Cambridge Isotope Laboratories, Inc.). They were allowed to grow for 5 h in the light with gentle stirring in a tightly closed flask. A 50-ml aliquot was harvested by centrifugation, and subjected to Bligh-Dyer partitioning. Lipids and starch (as part of insoluble materials) were recovered as described above. The remaining part of the culture was washed by 2 cycles of centrifugation and resuspension in fresh medium, and finally resuspended in 250 ml of fresh medium. The cells were allowed to grow under the photoautotrophic conditions for 4 days (Figure 1B). A 50-ml aliquot was harvested each day, and lipids and starch were recovered and stored as described. The experiments were performed in triplicates.

### Analysis of Lipids and Fatty Acids

All analytical methods (except ^13^C analysis) were essentially identical to those described previously (Sakurai et al., 2014; Toyoshima and Sato, 2015). Briefly, lipid classes were separated by two-dimensional thin-layer chromatography (2D-TLC). Each spot of lipid class was scraped off the plate, and subjected to methanolysis in 2.5% HCl in anhydrous methanol (Kanto Kagaku, Ltd., Tokyo, Japan) at 85°C for 2.5 h. Pentadecanoic acid (15:0) was added before methanolysis as an internal standard for quantitation of fatty acid methyl esters (FAMEs). After the reaction, FAMEs were extracted with *n*-hexane. Lipids were quantified as the amount of fatty acids analyzed as methyl esters by gas chromatography-mass spectrometry (GC-MS; model GCMS-QP2010 Ultra; Shimadzu, Kyoto, Japan) using a BPX70 column (length, 60 m; internal diameter, 0.22 mm; SGE Analytical Science, Victoria, Australia). Analytical conditions were described in a previous report (Sato et al., 2016). Mass spectra were used in two ways in the following analyses: First, total ion intensity was used to quantify the amounts of fatty acids using the internal standard. Second, mass distribution of molecular ion (M^+^) of FAME was obtained as a text file for the analysis of isotopomer distribution. For monoenoic fatty acids, the cluster of signals representing (M−31)^+^ and (M−32)^+^ was used for isotopomer analysis, while less intense peaks of M^+^ were used to check unrelated or contaminant fragments.

### Analysis of Starch

The insoluble fraction recovered from the fluff, containing starch, cell wall, among others, was dried under vacuum and then subjected to methanolysis in 2.5% HCl in anhydrous methanol to yield 1-methyl glucoside. Mannitol was added before methanolysis as an internal standard for quantitation. Hydrophobic materials were removed by extraction with *n*-hexane. After drying, 1-methyl glucoside was mixed with 20 μl of a trimethylsililation reagent, TMS-HT kit (Tokyo Chemical Industry CO., Ltd., Tokyo, Japan), and heated at 95°C for 10 min. The resulting tetrakis(TMS) 1-methyl glucoside was analyzed by GC-MS using an Rtx-5MS column (length, 30 m; internal diameter, 0.25 mm; Restek, Bellefonte, PA, United States). Analytical conditions were described in a previous report (Sato, 2015). Amount of glucose was quantified by the total ion intensities of two major peaks of tetrakis (TMS) 1-methyl glucoside using the internal standard. Mass distribution of the ion retaining all six carbons with four TMS groups (the mass of monoisotopic ion is 361) was obtained as a text file, and used for isotopomer analysis. As the mass distribution was essentially identical in the two peaks, the data of both peaks were averaged and used for the subsequent calculation.

As the cell wall of *Chlamydomonas* does not contain cellulose, but consists of glycoproteins with arabinose, mannose, galactose and glucose (Harris, 2009), we checked a possible interference by cell wall materials in the starch analysis by finding non-glucose sugars in the GC-MS chromatogram, but we did not detect notable levels of pentoses and hexoses other than glucose. Accordingly, we can safely consider that the glucose that we analyzed was derived from starch, but not from other materials.

### Isotopomer Analysis

Isotopomer analysis of glucose and fatty acids was performed essentially according to our original method using the C13dist software^1^ (Sato et al., 2016). For technical details, see the Appendix of Sato et al. (2016). Actual procedure that was adapted for this study is explained in Results (section “Overall Design of Labeling Experiments”) for readers unfamiliar with isotopomer analysis.

## Results

### Overall Design of Labeling Experiments

We performed two types of labeling experiments: in the first series of experiments (Figure 1A), the cells of *C. debaryana* were grown under the standard autotrophic conditions, and the cells at 48, 96, and 144 h were photosynthetically labeled with [^13^C]bicarbonate for 2 h, respectively. In the second series of experiments (Figure 1B), the cells grown for 72 h after inoculation was labeled with [^13^C]bicarbonate for 5 h under standard growth conditions. The sample at this point was called “L5h.” After washing, the cells were resuspended in fresh medium and then allowed to grow for further 4 days. Samples were taken at 1, 2, and 4 days, and named “C1d,” “C2d,” and “C4d,” respectively. Similar labeling and chase experiments were performed with [2-^13^C]acetate.

**Figure 2:**
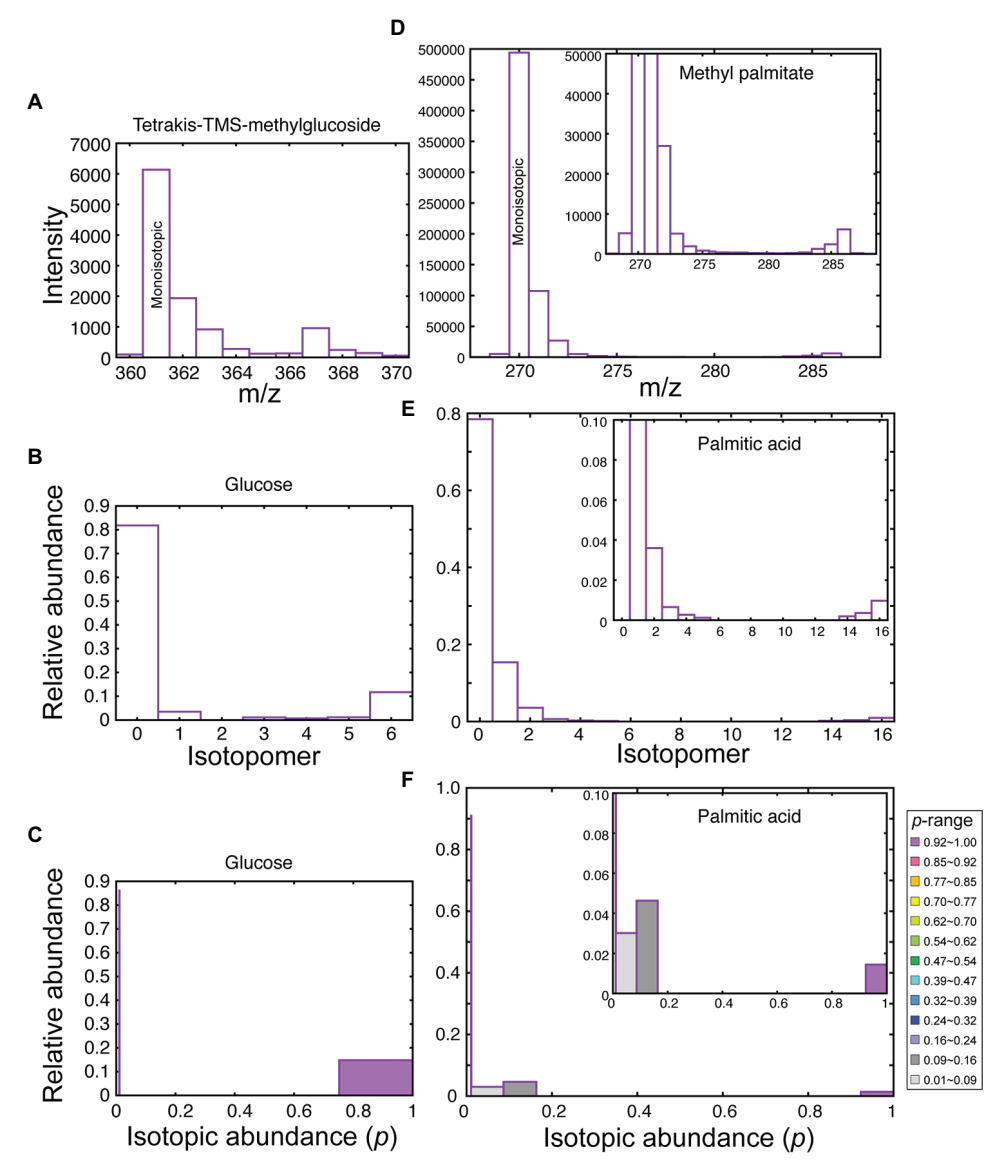
Data processing in isotopomer analysis. **(A)** Mass spectrum of the fragment ion having the monoisotopic *m/z* = 361 from tetrakis (TMS) methylglucoside (from starch after 2-h labeling with [^13^C]bicarbonate at the exponential growth phase, 48 h after innoculation). **(B)** Isotopomer distribution of glucose. **(C)** Distribution of isotopic abundance, estimated according to the band model of C13dist software, using four steps having the width of 0.247 plus the natural abundance (*p* = 0.0108) population. **(D)** Mass spectrum of the molecular ion of methyl palmitate (from TAG after 5-h labeling with [^13^C]bicarbonate followed by 4 days chase). **(E)** Isotopomer distribution of palmitate. **(F)** Distribution of isotopic abundance, estimated according to the band model, using 14 steps having the width of 0.0707 plus the natural abundance population. Inset in **(D–F)** shows an enlargement.

Starch was transmethylated to 1-methyl glucose, which was then analyzed by GC/MS as trimethylsilyl ether. Individual classes of lipids were isolated by 2D-TLC, and then the component fatty acids were analyzed by GC-MS as FAMEs. Two types of data were obtained from these analyses: first, the amounts of glucose or fatty acids were quantified using internal standard. Second, mass distribution of molecular or high-intensity ion was determined for isotopomer analysis (see Figure 2 as examples). Methyl glucoside was analyzed as TMS derivatives, and the fragment *m/z* = 361 retaining all six carbons with four TMS groups was used for isotopomer analysis (Figure 2A). Two isomeric peaks were found in the GC/MS analysis, but both peaks were used for the calculation because they gave similar mass spectra with respect to the fragment that we focused on. For FAMEs, molecular ion was used for isotopomer analysis (Figure 2D), except for monoenoic FAMEs, in which the pair of fragments (M-31)^+^ and (M-32)^+^ were used for the analysis, because their intensities were higher than that of the molecular ion. In all cases, isotopomer distribution was estimated from the raw mass spectrum by the C13dist software (Figure 2, panels B and E). For the technical details of the program, see Appendix in Sato et al. (2016). We also calculated isotopic abundance (*p*) distribution using the band model (the model 4 in the mode 5 in the C13dist software), namely, we created a step-wise model of *p*-distribution that best simulates the actual isotopomer distribution. We used four steps plus unlabeled population (five variables in total) for the analysis of glucose, and 14 steps plus unlabeled population (15 variables in total) for the analysis of C16 and C18 fatty acids. The selection of number of steps depended on the number of carbon atoms (*n*). The degree of freedom of the isotopomer data was *n* + 1. Least square fitting requires that the number of variables should be less than *n* + 1. This was the reason why the number of steps was different in glucose and fatty acids.

In this way, we were able to distinguish high-*p* population and unlabeled (*p* = 0.0108, natural abundance or *NA*) population in glucose (Figure 2C). Likewise, in fatty acids, we identified high-*p* population (purple in Figure 2F), as well as low-*p* population (gray) and very-low-*p* population (light gray) within the labeled pool of molecules. Mid-*p* population (red) was also found in particular cases (see below). The isotopomers 0, 1, and 2 in Figure 2E seemed to be a part of unlabeled molecules (with natural abundance) at a glance, but the amounts of isotopomers 1, 2, and 3 were higher than in the authentic unlabeled molecules. This gives the gray peaks (with low *p* values) in Figure 2F after fitting with the band model.

The *p* value is an intrinsic value of average ^13^C abundance in the respective pool of molecules. Namely, the pools with different values of *p* represent different pools. Such distinction is an advantage of using a stable isotope, which is not possible in radiolabeling experiments. Note, however, not all levels in the *p*-distribution graph represent different pools. The 5 or 15 levels were used to maximize the resolution of *p*. Metabolically meaningful pools were identified by the dynamics of individual populations assigned to the levels.

### Lipid Profile in Exponential and Stationary Growth Phases

**Figure 3:**
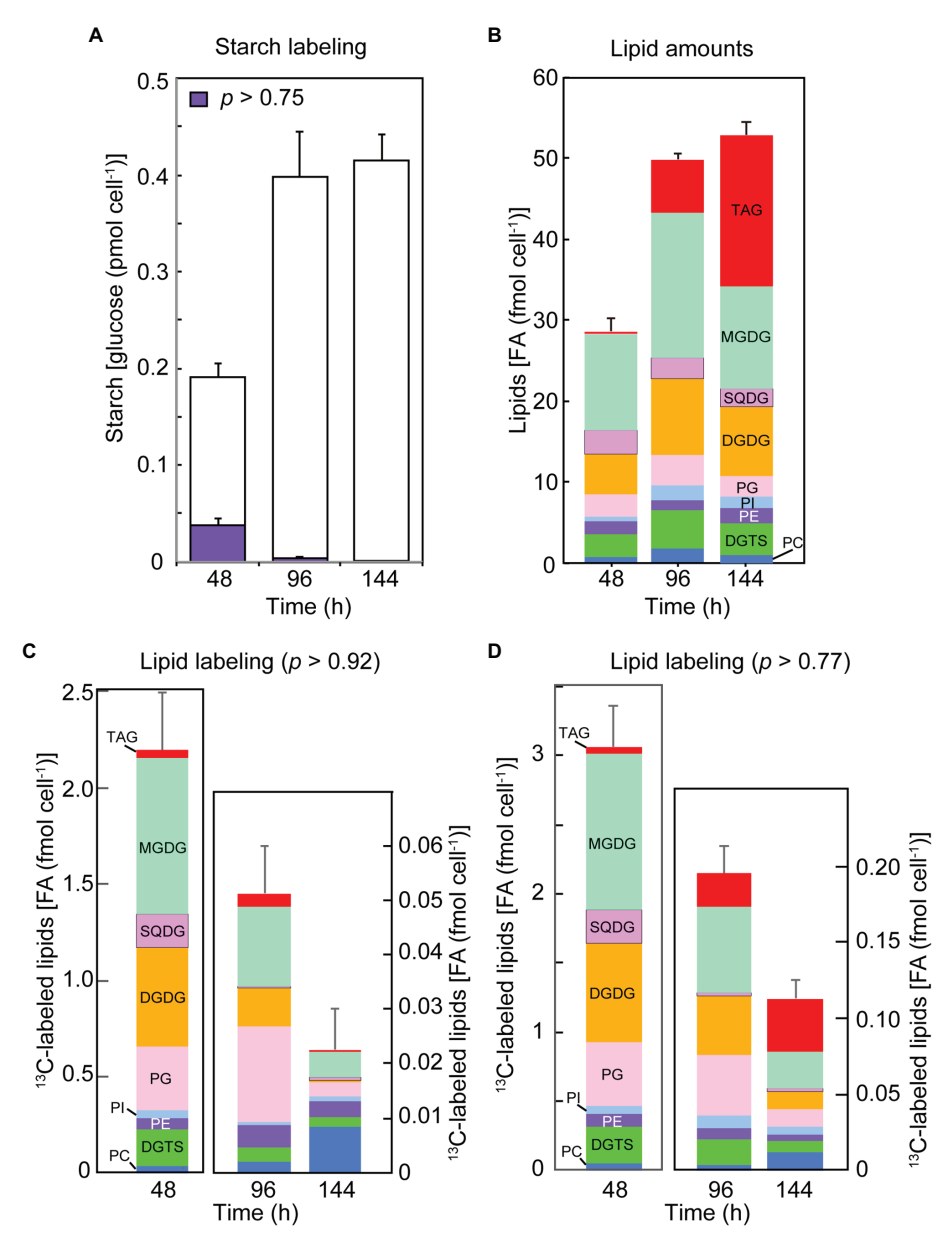
The contents of total and labeled fractions of starch and lipids after the labeling for 2 h with sodium [^13^C]bicarbonate at different growth stages. **(A)** The amounts of total and labeled (*p* > 0.75) fraction of starch. White bars show unlabeled glucose, while purple bars show labeled glucose. **(B)** The amounts of lipid classes. **(C)** The amounts of labeled lipids (*p* > 0.92). **(D)** The amounts of labeled lipids (*p* > 0.77). Lipid classes are color-coded. Each value represents the mean ± SE of three independent experiments.

The amounts of starch and lipids in exponentially growing cells (48 h), and early (96 h) and late (144 h) stationary phase cells are shown in Figures 3A,B, respectively. Note that the starch data also include labeling results, which will be a topic in later sections. The cellular contents of both starch and lipids increased in the stationary phase. Proportion of individual lipid classes did not change markedly, except extensive accumulation of TAG in the late stationary phase. Other major lipid classes were: monogalactosyl diacylglycerol (MGDG), sulfoquinovosyl diacylglycerol (SQDG), digalactosyl diacylglycerol (DGDG), phosphatidylglycerol (PG), phosphatidylinositol (PI), phosphatidylethanolamine (PE), diacylglyceryl-*N,N,N*-trimethylhomoserine (DGTS), and PC.

**Figure 4:**
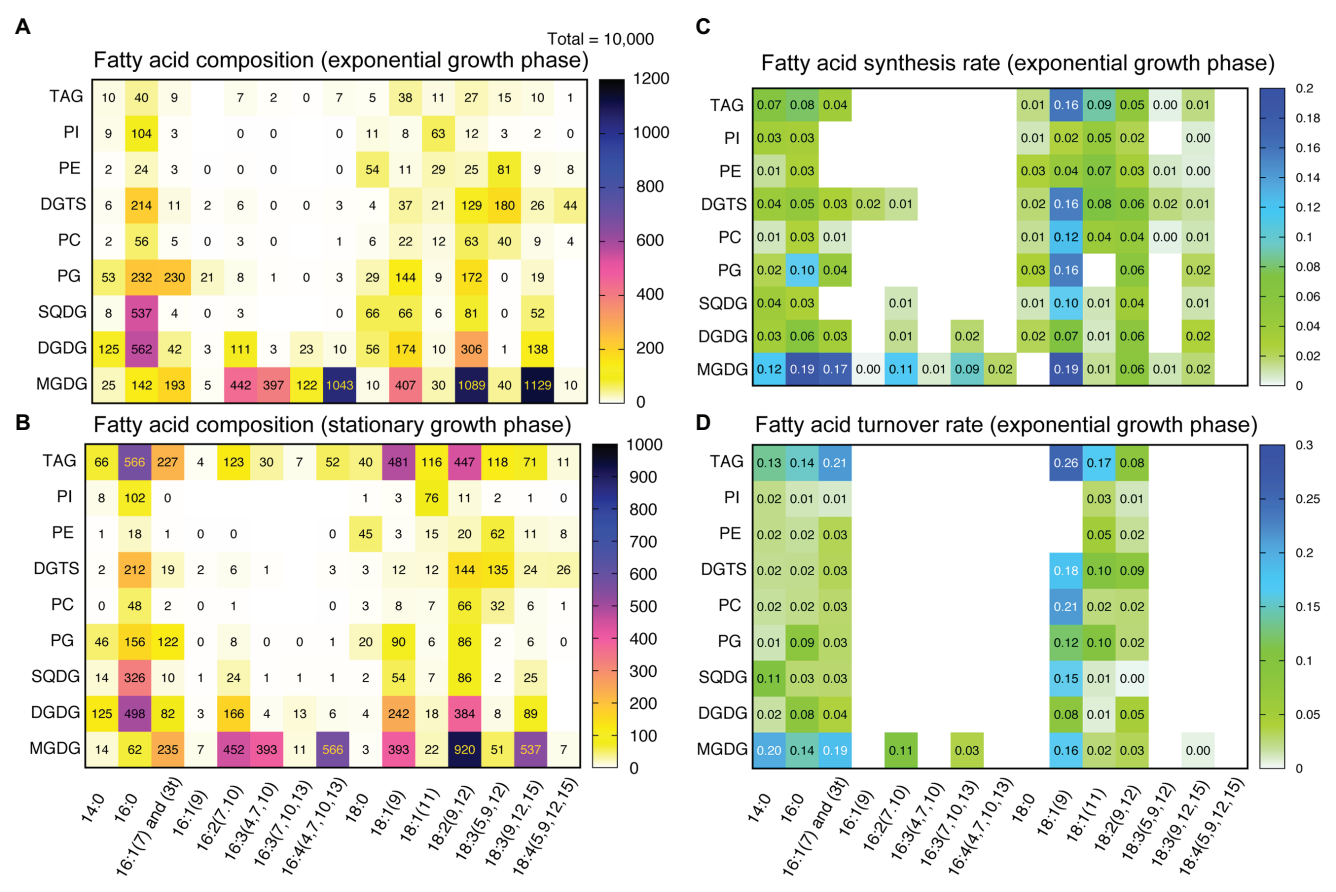
Fatty acid composition and fatty acid turnover rates in individual lipid classes. **(A)** Fatty acid composition of lipid classes in the exponential growth phase (48 h). **(B)** Fatty acid composition of lipid classes in the stationary growth phase (144 h). **(C)** Fatty acid synthesis rates of individual lipid classes. The rates (in h^−1^) were calculated for the data of 2-h labeling and 5-h labeling with [^13^C]bicarbonate according to Supplementary Material and averaged. **(D)** Fatty acid turnover rates of individual lipid classes calculated for the chase data after the labeling with [^13^C]bicarbonate according to Supplementary Material. In **(A,B)** contents of fatty acids are normalized by setting the amount of total fatty acids (of all lipid classes) to 10,000.

Figure 4 shows a heat-map representation of a concise overview of fatty acid composition in individual lipid classes in exponential (panel A: 48 h after inoculation) and stationary (panel B: 144 h after inoculation) growth phases. Major fatty acids were myristic (14:0), palmitic (16:0), hexadecenoic (16:1), oleic [18:1(9)], linoleic [18:2(9,12)], and α-linolenic [18:3(9,12,15)] acids in most lipid classes. MGDG also contained unsaturated C16 acids, such as 16:2, 16:3, and 16:4. Pinolenic [18:3(5,9,12)] acid was enriched in DGTS and PE. In the stationary phase, highly accumulated TAG contained 16:0, 16:1, 18:1(9) and 18:2, as well as various minor fatty acids found in polar lipids. Fatty acid profiles of other lipid classes were more or less similar in the exponential and stationary phases.

The analytical results of the lipid and fatty acid compositions were consistent with the results presented earlier in *C. debaryana* (Toyoshima and Sato, 2015, 2018; Yoshitomi et al., 2020).

### *De novo* Synthesis of Starch and Lipids

To measure the rate of *de novo* synthesis of starch and lipids, the cells of *C. debaryana* at different times of growth were allowed to incorporate ^13^C photosynthetically from sodium [^13^C]bicarbonate (*p* = 0.99) for 2 h (Figures 3A,C,D). In the labeling experiments with bicarbonate, the distribution of *p* was estimated from the observed isotopomer distribution.

In both exponential growth phase (48 h after inoculation) and early stationary growth phase (96 h), we found two populations of glucose in starch, one with ^13^C at the natural abundance level (1.08% or *NA*) and another labeled with ^13^C peaking at an average *p* = 0.97–0.98 (*p* > 0.75 in the four-step grading in the band model, but the actual distribution of *p* was also estimated by the C13dist program using the average model), which represented *de novo* synthesis resulting from photosynthetic carbon fixation (Figure 3A). As described already, we used the band model with five variables (four steps and an unlabeled population) for quantitation of differently labeled populations. In the 2-h labeling experiments, only the unlabeled and the high-*p* (*p* > 0.75) populations were observed. The labeled population amounted to 19.9% of glucose in starch in the exponential phase, but lowered in the early and late stationary phases (96 and 144 h) to 1.1 and 0.1%, respectively. This indicated a drastic decrease in photosynthetic starch production in the stationary phase. However, high-level accumulation of starch in the stationary phase suggested that starch could be synthesized from existing carbon source within the cell.

Lipid synthesis rate was estimated as the rate of ^13^C incorporation into component fatty acids. We determined the proportion of ^13^C abundance populations (14 steps plus the *NA* level, as shown in Figure 2F) in all major fatty acids in individual lipid classes, and summed the amount of each population over all these fatty acids. In this way, we present here the amount of labeled lipids with *p* > 0.92 and *p* > 0.77 during the 2-h labeling time (Figures 3C,D, respectively). The population with *p* > 0.92 represents the product of *de novo* photosynthesis, whereas the population with 0.77 < *p* ≤ 0.92 could result from a mixed precursor pool with photosynthates (with a high *p* value) and recycled carbon (with ^13^C at the *NA* level). The amounts of labeled population of lipids estimated by the two criteria were similar in the exponential phase, whereas the two values differed considerably in the early and late stationary phases, suggesting that lowered rates of photosynthesis (more than about 10-fold in Figure 3D) resulted in imperfect incorporation of labeled carbon into long-chain fatty acid products.

In the exponential phase, the composition of labeled lipids was similar to the composition of lipids accumulated at that time. This suggests that all classes of lipids were synthesized in parallel by the supply of newly synthesized fatty acids. In the stationary phase, especially at 144 h, the labeling of chloroplast lipids, such as MGDG, DGDG, and SQDG, was drastically decreased, whereas the labeling of PE and PC dominated. This could represent acyl recycling rather than *de novo* synthesis of the phospholipids. The *de novo* synthesis of TAG (*p* > 0.92; Figure 3C) also lowered in the stationary phase, but the relative activity of TAG synthesis became higher in the population with *p* > 0.77 (Figure 3D), suggesting that both new and recycled carbons contributed to the production of TAG.

The rates of synthesis of fatty acids in individual lipid classes were estimated from the results of labeling for 2 and 5 h (Figure 4C). The data of 5-h labeling were taken from the pulse-chase experiments described in the next section. Two major features are apparent: first, oleic acid [18:1(9)] was rapidly synthesized and incorporated into many classes of lipids, such as MGDG, PG, DGTS, PC, and TAG. Second, both C16 and C18 fatty acids in MGDG were rapidly synthesized. Unsaturated fatty acids, such as 16:1(7), 16:2(7,10), 16:3(7,10,13) and 18:2(9,12) were also labeled rather rapidly, suggesting that the desaturation of both C16 and C18 acids proceeds rapidly. This was consistent with the observation that the pools of desaturation intermediates were small (Figure 4A).

An interesting point to note is that DGDG was labeled efficiently in the exponential growth phase. This was evident in the amount of labeled DGDG as a whole (Figure 3C) and in the synthesis rates of various fatty acids in DGDG (Figure 4C). The distribution of labeled fatty acids in DGDG was similar to that of MGDG except that the labeling rate was about 1/3. This is a curious observation that we never found in other photosynthetic organisms, and will be discussed later.

### Labeling and Chase Experiments: Overview

The flow of carbon in starch and TAG was analyzed by pulse-labeling for 5 h and chase for 4 days. Both sodium [^13^C]bicarbonate and sodium [2-^13^C]acetate were used in the labeling. Bicarbonate was expected to be incorporated into all organic compounds, whereas acetate was expected to be mainly incorporated into fatty acids. Here, the results with bicarbonate will be analyzed in detail, because the resolution of isotopomer analysis was limited in the acetate labeling experiments (note that a fatty acid is composed of C2 units resulting from acetate, and only one of two carbons are labeled). The results with acetate were mainly used to confirm the results with bicarbonate.

**Figure 5:**
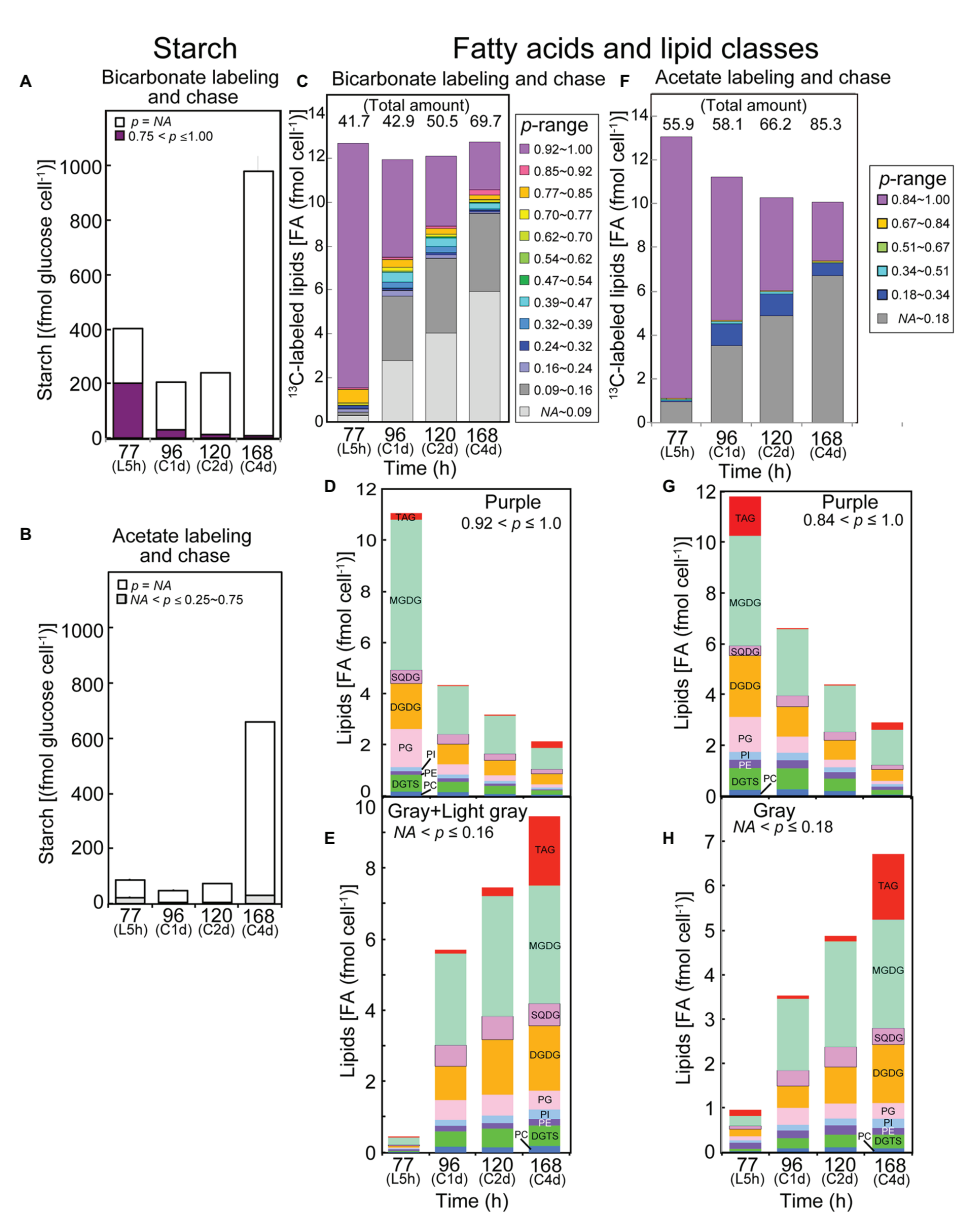
Analysis of labeled starch and lipids in the labeling and chase experiments with [^13^C]bicarbonate and [2-^13^C]acetate. **(A,C–E)** Bicarbonate labeling; **(B,F–H)** Acetate labeling. **(A,B)** Labeled and unlabeled starch. **(C,F)** Composition of isotopic abundance populations in total fatty acids. The amount of total lipids (as labeled plus unlabeled fatty acids) is shown on top. **(D,G)** Amounts of highly labeled lipid classes [purple population in **(C,F)**, respectively]. **(E,H)** Amounts of low-*p* lipid classes [gray + light gray, or gray populations in **(C,F)**, respectively]. Note that the number of steps in isotopic abundance is different in bicarbonate labeling and acetate labeling, because the maximum number of labeled carbons is different in the two experiments.

In the [^13^C]bicarbonate experiments, the content of high-*p* starch (*p* > 0.75) decreased significantly during the chase period, whereas the content of total starch increased (Figure 5A). In the [2-^13^C]acetate experiment, starch was not labeled to a significant level (Figure 5B). Namely, only a small proportion of starch glucose incorporated ^13^C from acetate into the low-*p* population (*NA* < *p* ≤ 0.25).

Figure 5C shows the amounts of variously labeled populations of lipids (exactly, their fatty acids) in the [^13^C]bicarbonate experiments. The total amount of lipids is described on top of each bar. Note that the unlabeled population (*p* = *NA*) is not explicitly shown in the figure (it is obtained by subtracting the labeled amount shown by the height of the bar from the total amount described at the top). The total amount of differently labeled populations did not change with chase time, but the content of high-*p* lipids (*p* > 0.92, purple part at the top of each bar) significantly decreased during the chase time. In contrast, the content of very low-*p* lipids (*NA* < *p* ≤ 0.16, the light gray and gray parts at the bottom of each bar) increased with time. This could reflect a dynamic turnover, either conversion of starch to lipids or degradation and re-synthesis of lipids, during the chase time. Comparable results were obtained in the experiments with [2-^13^C]acetate. Namely, high-*p* lipids (*p* > 0.84, top part in purple of each bar) decreased while low-*p* lipids (*NA* < *p* ≤ 0.18, bottom part in gray of each bar) increased (Figure 5F). Note that six steps of populations plus the unlabeled population was used in the analysis after [2-^13^C]acetate labeling, which restricted resolution of populations with different *p*-values. The results with acetate had to be understood within the framework of degradation and re-synthesis of lipids, because starch was not likely to act as a high capacity source with a high-*p* value (Figure 5B).

### Labeling and Chase Experiments: Bicarbonate Labeling

In the bicarbonate-labeling experiments, the rapid decrease of highly labeled starch and fatty acids (primary products of photosynthesis) during the chase period, and the concomitant increase of low-*p* population of fatty acids suggest that the degraded starch and fatty acids could be recycled in the synthesis of fatty acids. The carbon flow of re-synthesis must be fairly large. Judging from the data in Figure 5C, 6.65 fmol cell^−1^ (60%) of highly labeled fatty acids within the total of 11.09 fmol cell^−1^ were degraded within a day, and 5.27 fmol cell^−1^ (equivalent to 79% of the degraded fatty acids) poorly labeled fatty acids were re-synthesized. In the re-synthesis, only a small part (less than 10%) of carbon resulting from degradation of highly labeled fatty acids must be used because the *p* value of re-synthesized fatty acids was quite low (about 0.1). In other words, one part of carbon originating from degraded fatty acids (plus starch) is mixed with nine parts of newly fixed carbon to form a poorly labeled population with *p* = 0.1, which was used in *de novo* synthesis of fatty acids again.

**Figure 6:**
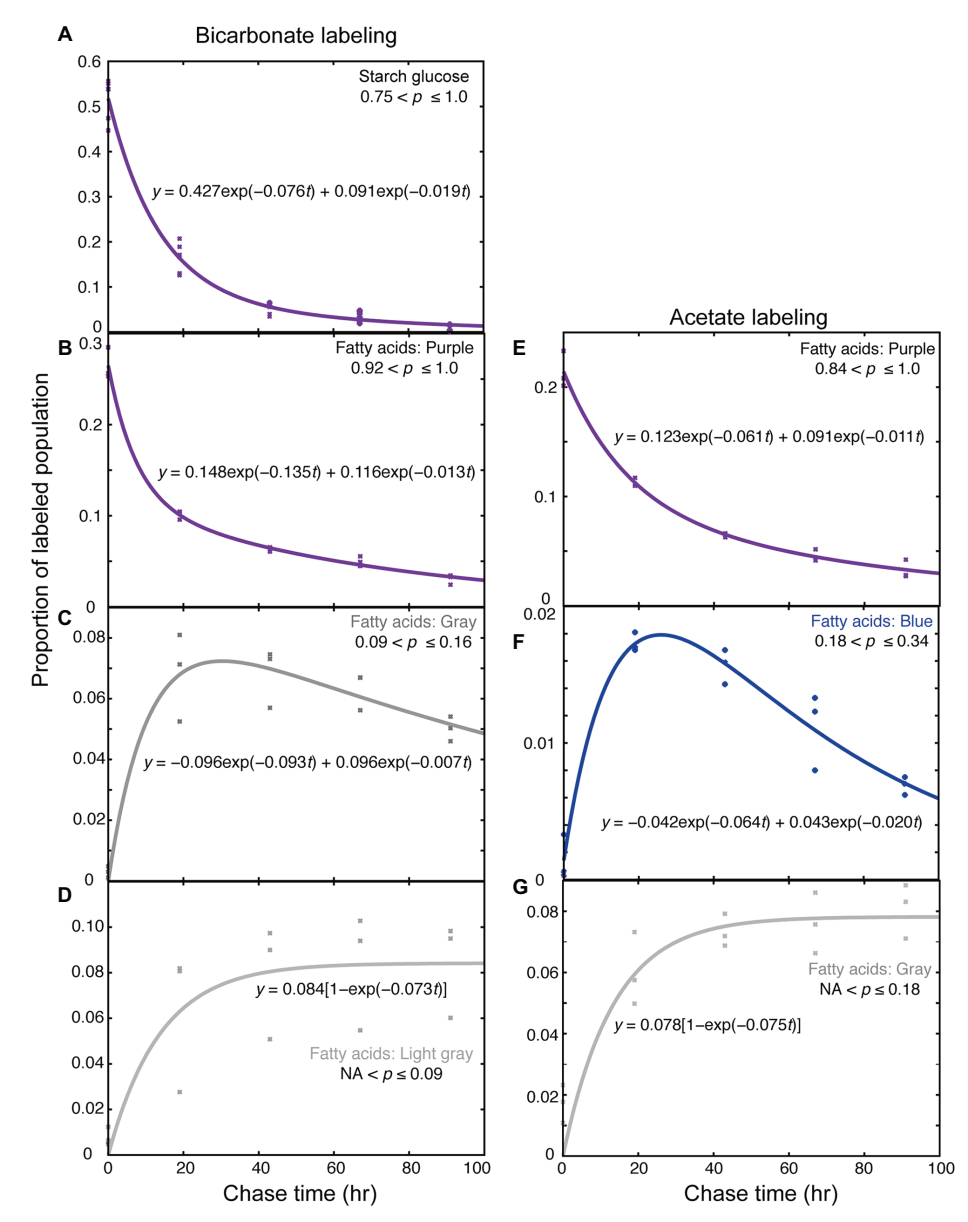
Kinetic analysis of labeled populations during the chase. **(A–D)** Labeling with [^13^C]bicarbonate. **(E–G)** Labeling with [2-^13^C]acetate. **(A)** Starch glucose; **(B–E)** Highly labeled fatty acids; **(C)** Low-*p* (gray population) fatty acids; **(D)** Very low-*p* (light gray population) fatty acids; **(F)** Mid-low-*p* (blue population) fatty acids; and **(G)** Low-*p* (gray population) fatty acids. Kinetic analysis was performed with the R software according to Supplementary Material.

The time course of changes in labeled population was analyzed kinetically. According to the theoretical treatment described in Supplementary Material, each time course consisting of a triplicate was fitted with a linear combination of two exponentials. The coefficients in the two exponents represented time constants of rapid and slow components, respectively. The slow components having *k* values of about 0.01–0.02 were likely to represent net degradation. The major decay rate *k* of starch (purple fraction) was 0.0759 ± 0.0285 h^−1^ (Figure 6A, average ± SE), while that of fatty acids (purple fraction) was 0.135 ± 0.067 h^−1^ (Figure 6B). Similar analysis was performed for each major fatty acid in individual lipid classes, but statistically meaningful results (*n* = 3) were obtained only for the fatty acids that have incorporated high level of ^13^C during the 5-h labeling. The decay rates of purple fraction (Figure 4D) were: 16:0 in MGDG: *k* = 0.138 ± 0.016, 18:1 in MGDG: *k* = 0.159 ± 0.371, 18:1 in DGDG: *k* = 0.083 ± 0.034, 18:1 in SQDG: *k* = 0.151 ± 0.027, 18:1 in PG: *k* = 0.122 ± 0.005, and 18:1 in DGTS: *k* = 0.182 ± 0.066. Figure 4D shows all values estimated in this way, including values with large estimated SE. Note that the labeling kinetics of 16:0 and 18:1 includes synthesis, desaturation, and degradation, but desaturation is compensated within total fatty acids. Therefore, the labeling kinetics of total fatty acids consists of only synthesis and degradation, and is simpler to analyze. Nevertheless, the turnover rates of fatty acids in individual lipid classes were roughly consistent with the corresponding synthesis rates (Figure 4C), supporting that the assumption of steady-state kinetics was applicable.

The increase rate of the gray (0.09 < *p* ≤ 0.16) population of fatty acids (0.093 ± 0.040 h^−1^, Figure 6C) and that of the light gray (*NA* < *p* ≤ 0.09) population of fatty acids (0.073 ± 0.035 h^−1^, Figure 6D) were similar, suggesting that both of these two populations were synthesized partially using the carbon supplied by degraded starch (*k* = 0.075) rather than degraded fatty acids (*k* = 0.135). Because the increase rates for fatty acids were an average of all fatty acids of total lipids, this does not exclude a possibility that a specific pool of fatty acids could supply materials for *de novo* fatty acid synthesis yielding the gray and light gray populations of fatty acids.

### Labeling and Chase Experiments: Acetate Labeling

In the case of acetate labeling, too, we can calculate the carbon flow of re-synthesis. Using the data in Figure 5F, 5.39 fmol cell^−1^ (45%) of highly labeled fatty acids within the total of 11.93 fmol cell^−1^ were degraded and re-synthesized as 3.50 fmol cell^−1^ (equivalent to 65% of the degraded fatty acids) poorly labeled fatty acids. The proportion of re-synthesis is estimated to be lower than that in the bicarbonate labeling experiments, but, we can still imagine that a large fraction of newly synthesized fatty acids were degraded and re-used for the synthesis of fatty acids.

Kinetic analysis of the fatty acid labeling with [^13^C]acetate was performed in a similar way (Figures 6E–G). The decrease of the high-*p* population (purple; 0.84 < *p* ≤ 1.0) consisted of two components, namely, rapid decay (*k* = 0.061 ± 0.030) and slow decay (*k* = 0.011 ± 0.008; Figure 6E). The kinetics of the blue population (0.18 < *p* ≤ 0.34) was biphasic, consisting of increasing (*k* = 0.064 ± 0.025) and decreasing (*k* = 0.020 ± 0.006) components (Figure 6F). The increase rate of the gray population (*NA* < *p* ≤ 0.18) was *k* = 0.075 ± 0.022 (Figure 6G). These rate constants were roughly consistent with the corresponding rate constants obtained with [^13^C]bicarbonate labeling. However, the rapid decrease rate of the high-*p* (purple) population was significantly higher in the bicarbonate labeling than in the acetate labeling. The difference in the *p*-range definition of the “purple” population in the two experiments (0.84 < *p* ≤ 1.0 in acetate labeling, and 0.92 < *p* ≤ 1.0 in bicarbonate labeling) was not a reason, because the proportion in the “red” population (0.85 < *p* ≤ 0.92) was quite low in bicarbonate labeling. An explanation might be that the high concentration (20 mM) of bicarbonate was a good carbon source to support (or activate) cell metabolism, whereas acetate was a poor carbon source, at least in photoautotrophically grown cells. Accordingly, lipids and starch were synthesized in excess during the labeling with bicarbonate (see the total amounts in Figures 5A,C), and then degraded after the shift to normal growth condition with 1.0% CO_2_ to attain a steady state. Except this initial rate, the flow of carbon in the low-*p* populations of fatty acids was consistent in both acetate and bicarbonate labeling experiments.

### Labeling and Chase Experiments: Lipid Classes

Dynamics of lipid labeling was analyzed in individual lipid classes in both bicarbonate labeling (Figures 5D,E) and acetate labeling (Figures 5G,H). Figure 5D shows the amounts of fatty acids of high-*p* population (purple in the bar graphs of fatty acid labeling) in lipid classes. The results were qualitatively similar to those in 2-h labeling (Figure 3C), but the proportion of labeled MGDG was higher in Figure 5D. The high-*p* population with acetate labeling contained high proportion of TAG (Figure 5G), although the composition of other lipid classes was similar to that in Figure 5D (bicarbonate labeling for 5 h). This suggests that TAG synthesis was stimulated by the supply of high concentration of acetate (20 mM).

During the following chase period, all classes of lipids lost the high-*p* population more or less similarly, except that the labeled TAG that accumulated during the labeling was lost rapidly, and accumulated in the stationary phase again (Figures 5D,G). Conversely, the low-*p* population of fatty acids accumulated in all lipid classes during the chase period (Figures 5E,H). The accumulation pattern was similar in bicarbonate and acetate labeling. In the stationary phase, accumulation of TAG was marked.

**Figure 7:**
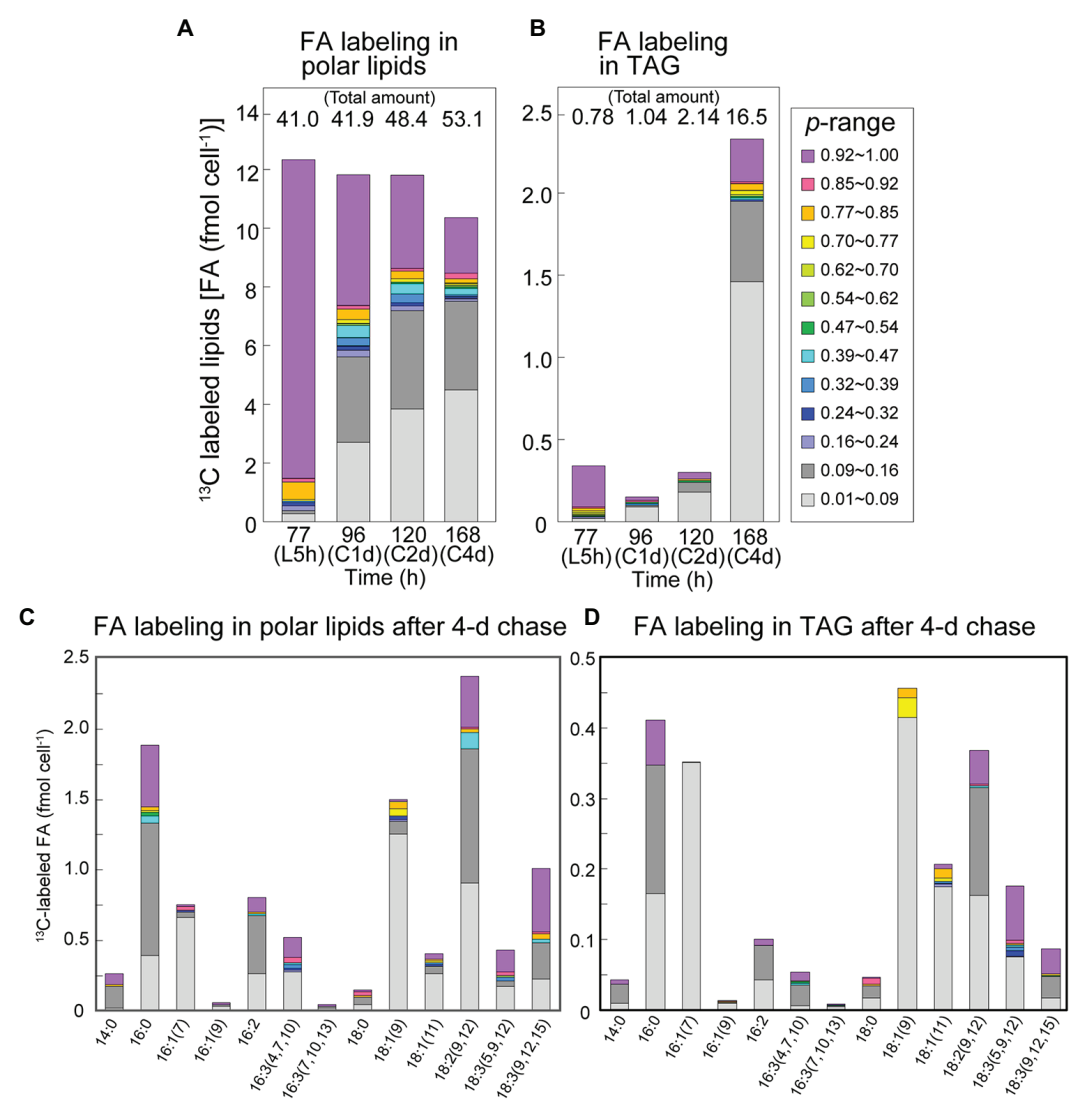
Fatty acid labeling in polar lipids and TAG in the [^13^C]bicarbonate labeling and chase experiments. **(A)** Composition of isotopic abundance populations in the fatty acids of polar lipids. **(B)** Composition of isotopic abundance populations in the TAG fatty acids. Total amount of lipids (as fatty acids) is shown on top. **(C)** Composition of labeled populations of fatty acids in polar lipids after the chase for 4 days. **(D)** Composition of labeled populations of fatty acids in TAG after the chase for 4 days.

Figure 7 shows changes in the isotopic abundance distribution of polar lipids and TAG after the labeling with bicarbonate and chase. The changes in polar lipids (Figure 7A) were similar to those in total lipids (Figure 5C), but we noted a decrease in labeled population with concomitant increase in the total polar lipids (including unlabeled lipids) in the stationary phase (48–53 fmol cell^−1^ from 120 to 168 h; Figure 7A). High-level accumulation of TAG was found in the stationary phase (16.5 fmol cell^−1^), and labeled population accounted for 2.4 fmol cell^−1^, about 80% of which consisted of populations with *NA* < *p* ≤ 0.16. The net increase in unlabeled fatty acids (14.1 fmol cell^−1^) in TAG was accounted for by *de novo* synthesis. Interestingly, high-*p* population (purple) was found in 16:0, 18:2, and 18:3 in TAG (Figure 7D). Very low-*p* population (light gray) was abundant in 18:1 and 16:1. This suggests that labeled 16:0, 18:2 and 18:3 were mobilized from polar lipids (compare Figures 7C,D) to TAG, whereas the labeled 18:1 and 16:1 in TAG were products of re-synthesis from carbons recovered from degraded labeled fatty acids with newly fixed carbons or internal unlabeled carbon source.

**Figure 8:**
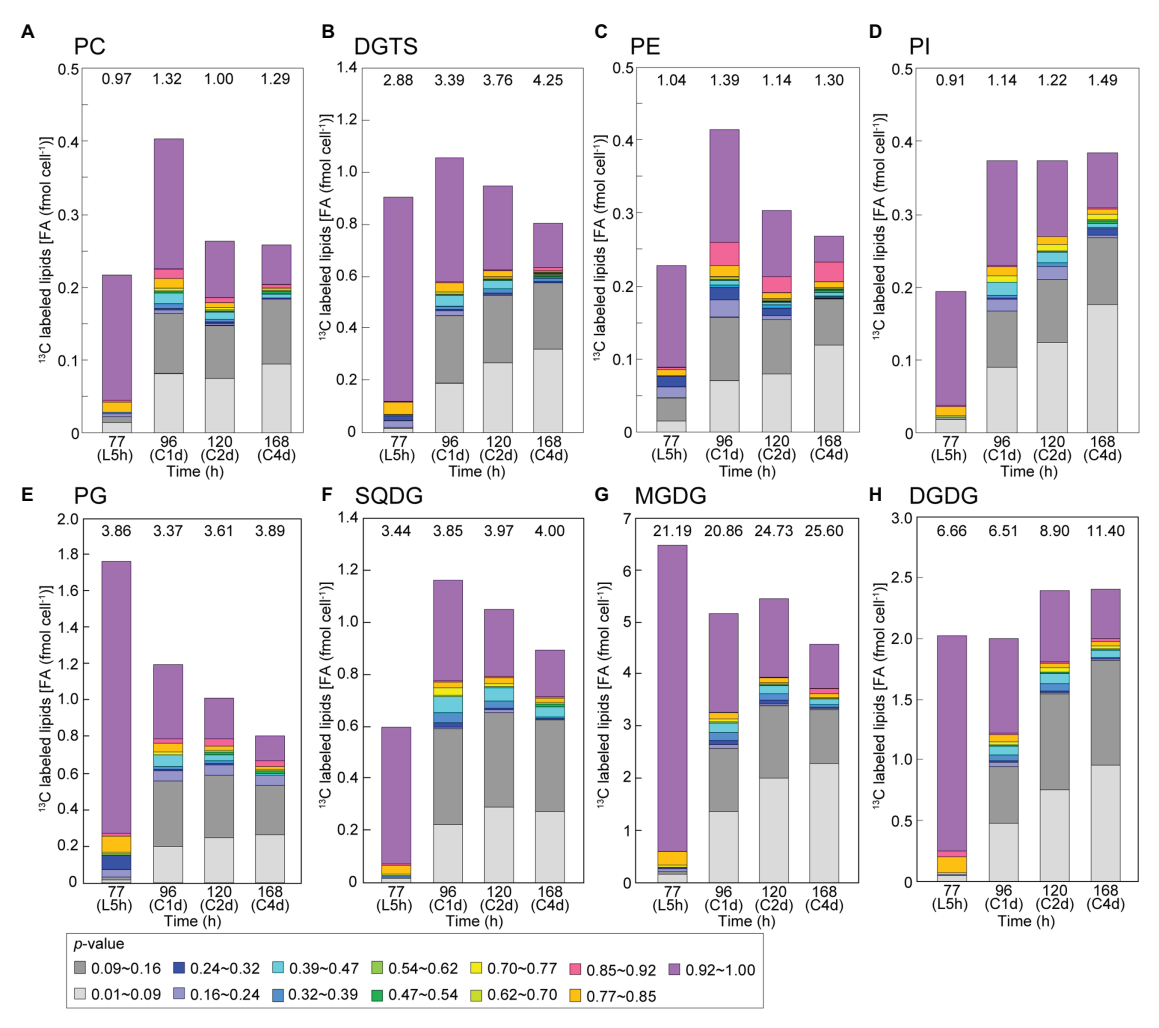
Composition of isotopic abundance populations in the fatty acids in individual classes of polar lipids in the [^13^C]bicarbonate labeling and chase experiments. **(A)** Phosphatidylcholine (PC); **(B)** diacylglyceryl-*N,N,N*-trimethylhomoserine (DGTS); **(C)** phosphatidylethanolamine (PE); **(D)** phosphatidylinositol (PI); **(E)** phosphatidylglycerol (PG); **(F)** sulfoquinovosyl diacylglycerol (SQDG); **(G)** monogalactosyl diacylglycerol (MGDG); and **(H)** digalactosyl diacylglycerol (DGDG). Total amount of lipids (as fatty acids) is shown on top.

**Figure 9:**
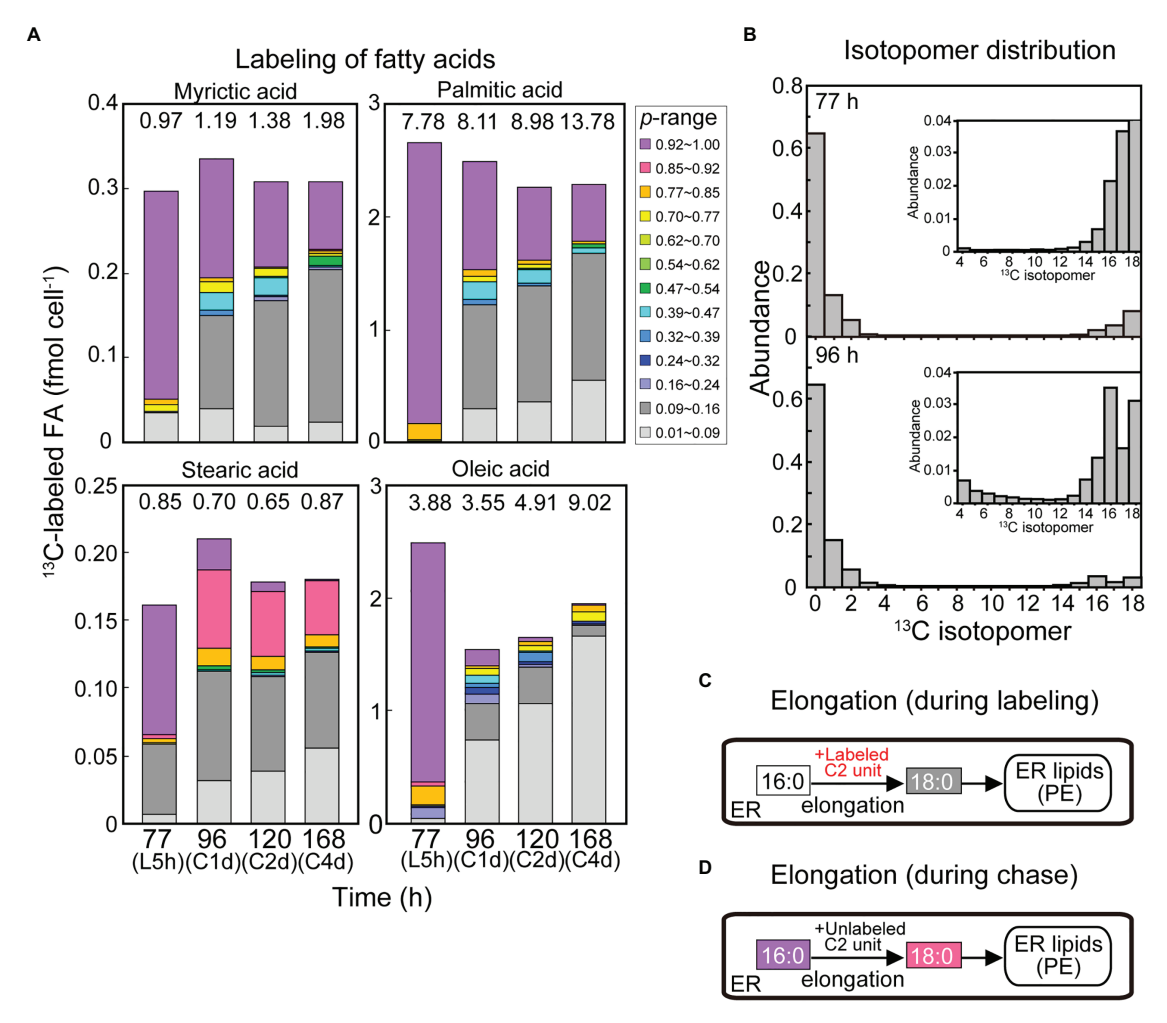
Curious labeling pattern in stearic acid in the [^13^C]bicarbonate labeling and chase experiments. **(A)** Composition of isotopic abundance populations in saturated fatty acids and oleic acid. **(B)** Isotopomer distribution of stearic acid after the labeling for 5 h and after the chase for 1 day. **(C,D)** Explanation of stearic acid labeling by elongation during the labeling **(C)** and the chase **(D)**.

Figure 8 shows composition of labeled populations in individual lipid classes in the bicarbonate-labeling experiments, namely, a decomposition of the data in Figure 7A. Clearly, the chloroplast lipids, MGDG, DGDG, SQDG, and PG contained the major part of high-*p* population (purple) of fatty acids after the 5-h labeling. The high rate of labeling of DGDG will be discussed later. Decrease in the high-*p* population and increase in low-*p* populations (gray and light gray) were found in all lipid classes during the chase. The net decrease in labeled fatty acids in MGDG and PG during the first day of chase was counter-balanced by the increase in labeled fatty acids in PC, DGTS, PE, PI and SQDG. Interestingly, the population shown in red corresponding to 0.85 < *p* ≤ 0.92 appeared at significant levels in PE after the first day of chase. This was, in fact, concentrated in 18:0 (Figure 9C).

Figure 9 shows labeled populations in saturated fatty acids and oleic acid. The composition of labeled populations in stearic acid (18:0) was quite different from that of other fatty acids. Namely, the proportion of purple population (0.92 < *p*) was lower (6.1% of total 18:0) and the proportion of gray population (0.09 < *p* ≤ 0.16) was higher (11.3% of total 18:0) compared with other saturated fatty acids after the labeling with [^13^C]bicarbonate for 5 h (Figure 9A). After the chase for first day onwards, the purple population diminished, whereas the red population (0.85 < *p* ≤ 0.92) as well as the gray population became abundant. Close examination of the isotopomer distribution (Figure 9B) showed that the isotopomer containing 16 atoms of ^13^C appeared after the chase, which represented the red population. The red population persisted until the fourth day of chase. As shown in Figure 8C, PE was the major lipid class that contained the red population of stearic acid. The red population (0.85 < *p* ≤ 0.92) was only a minor component in all other fatty acids.

## Discussion

### Methodological Advantage of Isotopomer Analysis

The present study was planned to exploit the possibility of stable isotope labeling in elucidating dynamism of carbon flux related to accumulation of starch and lipids. Detailed isotopomer analysis using C13dist software revealed both highly labeled population (*p* > 0.92) and low abundance population (*NA* < *p* ≤ 0.16) in fatty acids. Labeling and chase studies showed distinct pools with differently labeled populations. The low abundance population is likely to result from degradation of highly labeled population and re-synthesis with about 9-fold excess of unlabeled, newly fixed carbon. The two substrates, bicarbonate and acetate, provided distinct but compatible information: namely, both starch and lipids were efficiently labeled with [^13^C]bicarbonate, whereas only lipids were labeled significantly with [2-^13^C]acetate. Bicarbonate is a good tracer for general metabolic flow, but acetate was useful in fatty acids labeling.

### Turnover of Starch and Lipids: Labile and Stable Pools

**Figure 10:**
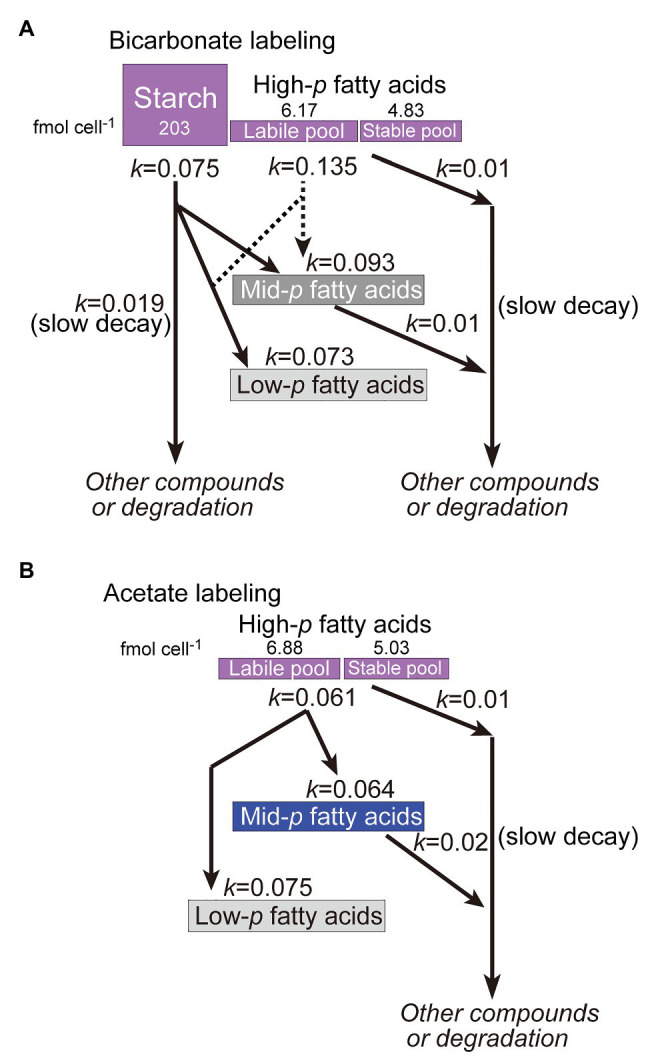
Model of labeling and remodeling of starch and fatty acids as deduced from the labeling and chase experiments. **(A)** Bicarbonate labeling; **(B)** Acetate labeling.

Kinetic analysis suggested that both starch and TAG were subject to dynamic metabolism during both exponential and stationary growth phases. The turnover rate (kinetic constants of 0.075–0.135 h^−1^, equivalent to half-lives of 9–5.1 h) was markedly higher than the growth rate (0.026 h^−1^ equivalent to a doubling time of 27 h). Abundant supply of ^13^C-labeled substrate seemed to promote synthesis of starch and TAG, which were rapidly converted to other substances including fatty acids of polar lipids. Starch and fatty acids are different in metabolic turnover. Labeled starch is rapidly turned over, and no stable pool remained. In contrast, fatty acids also turned over rapidly to some extent, but a stable pool remained even after 4 days. The rapid and slow components in the kinetic analysis (Figure 6) represent labile and stable pools, respectively (Figure 10). This indicates that starch is present as a single pool, whereas different pools of fatty acids (in lipids) exist.

Close examination of the results of isotopomer analysis suggested that fatty acid synthesis seems to proceed using two carbon pools: the direct product of photosynthesis and the internal carbon pool resulting from degradation of fatty acids and starch. Two lines of evidence support this view: The first was the presence of lipid population with 0.77 < *p* ≤ 0.92 (Figure 3, panels C and D) as pointed out previously (see section “*De novo* synthesis of starch lipids”). This suggests that newly fixed carbon is mixed with some internal carbon pool. Second, the conversion of the highly labeled population of fatty acids (shown in purple in Figures 5, 8) to the poorly labeled population (shown in gray and light gray) during the chase for 1 day showed a rapid turnover of newly synthesized fatty acids and re-synthesis. The degradation of highly labeled fatty acids results in highly labeled population of acetyl CoA, which is mixed with unlabeled acetyl CoA derived from photosynthetically produced glyceraldehyde 3-phosphate to re-synthesize fatty acids. This rapid remake is constantly functioning in this green alga. This is different from the situation in cyanobacteria that lack β-oxidation system (see Gclust database at http://gclust.c.u-tokyo.ac.jp/, Sato and Obayashi, 2021), in which highly labeled population of fatty acids remained stable for at least 1 day (Sato et al., 2016). The carbon flow of re-synthesis in *C. debaryana* must be fairly large, about 50% of the newly synthesized fatty acids were degraded within a day (see Figure 10 for the sizes of labile and stable pools). Fatty acid synthesis and β-oxidation are likely to form a cycle to flexibly modulate the amounts of lipids and the fatty acid composition of lipids. This means that an excess of carbon source is rapidly compensated by the synthesis of fatty acids, which are then targeted to TAG. The following decrease in carbon supply accelerated degradation of TAG for the synthesis of other materials. This flexibility of fatty acid metabolism is a basis of TAG accumulation in the stationary growth phase.

Interestingly, however, not all labeled fatty acids were degraded during the 4 days of chase. About 20% of the initially synthesized, highly labeled population of fatty acids persisted as labeled 16:0 and other fatty acids (19.4% in Figure 5C for all fatty acids, and 20.2% in Figure 9A for 16:0). This suggests that there were two different pools of fatty acids: one (labile pool) that was subject to rapid turnover, and another (stable pool) that remained stable for a fairly long time. Curiously, only labile pool was found in oleic acid: namely, the highly labeled population of oleic acid disappeared within 2 days of chase, and replaced by a very low *p*-population. The decay kinetics of oleic acid consisted of a single component (*k* = 0.134 ± 0.008).

Various ideas can explain the two different pools of fatty acids, labile and stable ones. A rough estimate suggests that the stable pool consists about 40% of total newly synthesized lipids (Figure 10). An idea is the presence of different cell populations or cell differentiation. However, it is difficult to imagine that fatty acids are stable in some population of cells, because oleic acid is entirely labile. Another simple idea is that chloroplast lipids are more stable, because degradation of fatty acids takes place in the peroxisomes. Nevertheless, the data in Figure 8 do not support this idea. Stable fatty acid pool is found in all classes of lipids. Stable fraction was found in 16:0, 18:2, 18:3(5,9,12) and 18:3(9,12,15). Another idea is that the stable lipids are localized to special sites. Alternatively, stable lipids are bound to membrane proteins. We might be able to explain the data by just assuming that some fraction of newly synthesized fatty acids are rapidly degraded and recycled, and some other fraction became stabilized. Mathematical modeling could be useful to check this metabolic dynamism in the future.

Metabolic relationship between starch and TAG has been studied in *C. reinhardtii* (Siaut et al., 2011; Singh et al., 2014; Smith and Gilmour, 2018). Because *C. reinhardtii* accumulates TAG only under nitrogen deprivation in these studies using Tris-acetate medium, acetate was also used for starch synthesis. This is an important difference from the present study, in which no nitrogen deprivation was imposed. As described above, acetate was a poor substrate for starch synthesis in *C. debaryana* under the nutrient replete condition. The relationship between starch and TAG could be different in the two species of *Chlamydomonas*. Siaut et al. (2011) did not find clear correlation between starch and TAG accumulations in various strains and mutants. This is consistent with our inability to provide evidence that starch is a direct precursor to TAG. Smith and Gilmour (2018) found a diurnal change in starch synthesis in the light period in the 12-h light/12-h dark cycles. Our findings suggest that starch is rapidly turned over even in the light. Therefore, the balance between synthesis and degradation, as well as nitrogen availability, should be important in evaluating the data in light/dark experiments. Singh et al. (2014) used ^13^C-NMR for analyzing the fate of ^13^C, supplied as [^13^C]acetate or [^13^C]bicarbonate. An important conclusion from the study was that acetate is degraded to bicarbonate/CO_2_^aq^ before incorporation into TAG. This is not consistent with our data, showing that acetate is directly used in TAG synthesis (Figure 5G). If [2-^13^C]acetate that we used were once degraded to inorganic carbon, then the synthesized fatty acids would have a different isotopomer distribution. Namely, palmitate could have isotopomers having more than eight ^13^C atoms as in the experiments with [^13^C]bicarbonate. However, our data showed only isotopomers 0–8 (mostly 8) in the palmitate after [2-^13^C]acetate labeling (found as a purple population). This is evidence for the direct use of acetate in fatty acid synthesis for TAG production. The discrepancy could be explained by differences in time scale. The NMR experiments were performed after 24 h labeling when added acetate was exhausted. Alternatively, their data could reflect rapid recycling of added acetate after incorporation into labile pool of fatty acids as described above. NMR could be a good tool for the analysis of isotope incorporation at the cellular level, but it is usually difficult to analyze the label in different parts of molecules. Mass spectral analysis of various compounds can provide more detailed information on the incorporation of the label.

### Source of TAG Accumulation in the Stationary Growth Phase

In the stationary growth phase, very high accumulation of TAG was observed. As stated above, *de novo* synthesis of TAG is also active in the stationary growth phase (Figure 3D), although the net synthesis of total lipids was reduced in the stationary growth phase. Close examination of the labeled populations in TAG showed that about 1/5 of TAG (Figure 7B) contained both highly labeled and low abundance population of fatty acids (16:0 and polyunsaturated fatty acids; Figure 7D), which indicated conversion of existing polar lipids to TAG. The presence of highly labeled population in 18:2 and 18:3 in both polar lipids and TAG (Figures 7C,D) can be explained by gradual progress of desaturation, in which 18:1 is desaturated to 18:2, and then to 18:3. Highly labeled population in 18:1 after the 5-h labeling was rapidly converted to 18:2 and 18:3, and remained as polyunsaturated fatty acids. The highly labeled population of 16:0 and 18:3(5,9,12) in TAG could originate from the corresponding population in DGTS (Figure 8B), which is a major lipid class containing 18:3(5,9,12) (Figures 4A,B) and estimated to be an important source of TAG as described in *C. reinhardtii* (Sakurai et al., 2014).

In a previous paper (Toyoshima and Sato, 2018), we found that accumulation of starch preceded accumulation of TAG, which suggested that starch was a source of TAG. In the present study, direct precursor-product relationship between starch and TAG was not identified, because labeled starch in the exponential phase was rapidly metabolized before the accumulation of TAG in the stationary phase. The relationship might be rather indirect, namely, starch is first converted to polar lipids, which is then converted to TAG in the stationary phase. This does not exclude the possibility that starch provides carbon for the synthesis of TAG as an unlabeled carbon flow in the stationary phase.

### Detection of Palmitic Acid Elongation *in vivo*

The results shown in Figure 9 are best explained by fatty acid elongation. We had two observations (Figure 9A): first, the abundance of gray population in 18:0 after the labeling for 5 h; second, the abundance of red population in 18:0 after the chase. The first observation can be explained by assuming that unlabeled 16:0 was elongated by the addition of a labeled C2 unit during the labeling period (Figure 9C). The second observation can be explained by the elongation of labeled 16:0 by the addition of an unlabeled C2 unit during the chase period (Figure 9D). In both cases, the substrate 16:0 could be supplied by an old pool already present in the cells either as free fatty acids or acyl lipids rather than by a pool of newly synthesized fatty acids. The added C2 unit could be supplied directly from photosynthesis, and represents the labeling status of the cells.

If this interpretation is correct, this is evidence for the elongation of fatty acids *in vivo*. Elongation of fatty acids, such as palmitic acid, is known to occur in the ER of eukaryotic cells. Elongation is characterized biochemically and genetically, but the *in vivo* evidence of elongation is a fortunate result of using this alga. In a red alga, *Cyanidioschyzon merolae*, 16:0 was found to be exported from the chloroplast and elongated to 18:0 in the ER (Mori et al., 2019). In plants, fatty acids are nearly exclusively synthesized within the chloroplasts (mitochondrial fatty acid synthesis is a very minor activity), in which 16:0 is elongated to 18:0, which is then desaturated to 18:1. In a typical view of plant fatty acid metabolism, the pool size of 18:0 (in the chloroplast) is very small, and 16:0 and 18:1 are either utilized in the chloroplast or exported to the cytoplasm. Elongation of both 16:0 and 18:1 results in the synthesis of very long chain fatty acids, such as C20 and C22, to even C30 depending on tissue. In *Arabidopsis*, the product of the *FATTY ACID ELONGATION1* (*FAE1*) gene extends the chain length of fatty acids from C18 to C20 and C22 (James et al., 1995; Millar and Kunst, 1997). In this context, the elongation of 16:0 to 18:0 in the ER is considered an initial step of synthesis of very long chain fatty acids, rather than a process of producing 18:0 *per se*. In contrast, the present study showed that the synthesis of 18:0 by elongation in the ER provides 18:0 in PE. This is a “late” reaction occurring after the synthesis of 16:0. In other words, the elongation occurs using a metabolically inert pool of 16:0, which stays several hours after its synthesis. The 18:0 in PE was rather stable during the chase, which also represents a distinct, stable pool.

### Rapid DGDG Labeling

Rapid labeling of DGDG was found in the 2-h labeling experiments (Figure 3) as well as the 5-h labeling step of labeling and chase experiments (Figure 5). In many organisms, such as cyanobacteria (Sato and Murata, 1982), algae (Sato and Moriyama, 2007; Mori et al., 2019, in *Cyanidioschyzon merolae*, Sato, 1991 in *Cryptomonas*), and plants (Williams et al., 1975; Heemskerk et al., 1991), the acyl groups of DGDG are not readily labeled in typical labeling experiments for 1 or 2 h. We already demonstrated that the outer galactose of DGDG is preferentially labeled in cyanobacteria (Sato and Murata, 1982) and red algae (Sato and Moriyama, 2007). DGDG is synthesized by galactosylation of MGDG, which precludes direct acylation of newly synthesized fatty acids to DGDG. The results in *C. debaryana* suggest that a pool of newly synthesized MGDG is efficiently converted to DGDG, because the turnover rates of acyl groups of DGDG were smaller than those of MGDG acyl groups (Figure 4D). Among the two possible galactosylation mechanisms of MGDG to DGDG, galactolipid-galactolipid galactosyltransferase (*Arabidopsis* SFR2) is present in only land plants (see the Gclust database: Cluster 10,083 in Gclust2012_42: Sato and Obayashi, 2021). *Chlamydomonas reinhardtii* is unlikely to possess it (Riekhof et al., 2005). Only DGD1 is likely to transfer a galactose to MGDG from UDPgalactose to synthesize DGDG in *C. debaryana* according to the draft genome data (Hirashima et al., 2016). Curiously, Moseley and Thompson (1980) presented a preliminary result showing the rapid labeling of DGDG in *Volvox carteri*, a close relative of *Chlamydomonas*. We still do not know if rapid labeling of DGDG is common in green algae.

### Concluding Remarks

In the present study, we performed labeling experiments with a stable carbon isotope coupled with detailed isotopomer analysis in *C. debaryana*, to find the mechanism of TAG accumulation in the stationary growth phase. Carbon metabolism involving starch and lipids was rather complex, and we found various aspects of metabolism in this green alga: namely, two pools for the *de novo* synthesis of fatty acids, rapid turnover of starch and fatty acids including rapid TAG turnover, rapid DGDG synthesis, elongation of existing palmitic acid, degradation and re-synthesis of fatty acids, presence of a stable pool of lipids, and remodeling of polar lipids to TAG. Rapid turnover of starch was likely to provide carbon for the synthesis of fatty acids, but direct conversion of starch to TAG was not supported. In summary, the present study confirmed the power of isotopomer analysis in revealing dynamics of metabolic flow.

## Data Availability Statement

The raw data supporting the conclusions of this article will be made available by the authors, without undue reservation.

## Author Contributions

All authors listed, have made substantial, direct and intellectual contribution to the work, and approved it for publication.

### Conflict of Interest

The authors declare that the research was conducted in the absence of any commercial or financial relationships that could be construed as a potential conflict of interest.

## Footnotes

^1^http://nsato4.sakura.ne.jp/old/C13dist.html

## References

[ref1] Aikawa S. Izumi Y. Matsuda F. Hasunuma T. Chang J. S. Kondo A. (2012). Synergistic enhancement of glycogen production in *Arthrospira platensis* by optimization of light intensity and nitrate supply. Bioresour. Technol. 108, 211–215. 10.1016/j.biortech.2012.01.004, PMID: 22277210

[ref2] Aikawa S. Joseph A. Yamada R. Izumi Y. Yamatishi T. Matsuda F.. (2013). Direct conversion of *Spirulina* to ethanol without pretreatment or enzymatic hydrolysis processes. Energy Environ. Sci. 6, 1844–1849. 10.1039/c3ee40305j

[ref3] Bates P. D. (2016). Understanding the control of acyl flux through the lipid metabolic network of plant oil biosynthesis. Biochim. Biophys. Acta 1861, 1214–1225. 10.1016/j.bbalip.2016.03.021, PMID: 27003249

[ref4] Bates P. D. Stymne S. Ohlrogge J. (2013). Biochemical pathways in seed oil synthesis. Curr. Opin. Plant Biol. 16, 358–364. 10.1016/j.pbi.2013.02.015, PMID: 23529069

[ref5] Bhowmick G. D. Koduru L. Sen R. (2016). Metabolic pathway engineering towards enhancing microalgal lipid biosynthesis for biofuel application—a review. Renew. Sust. Energ. Rev. 50, 1239–1253. 10.1016/j.rser.2015.04.131

[ref6] Bligh E. G. Dyer W. J. (1959). A rapid method of total lipid extraction and purification. Can. J. Biochem. Physiol. 37, 911–917. 10.1139/o59-099, PMID: 13671378

[ref7] Bréhélin C. Kessler F. van Wijk K. J. (2007). Plastoglobules: versatile lipoprotein particles in plastids. Trends Plant Sci. 12, 260–266. 10.1016/j.tplants.2007.04.003, PMID: 17499005

[ref8] Chapman K. D. Dyer J. M. Mullen R. T. (2012). Biogenesis and functions of lipid droplets in plants: thematic review series: lipid droplet synthesis and metabolism: from yeast to man. J. Lipid Res. 53, 215–226. 10.1194/jlr.R021436, PMID: 22045929 PMC3269164

[ref9] Harris E. H. (2009). The Chlamydomonas sourcebook. 2nd Edn. Vol. 1. Amsterdam: Academic Press.

[ref10] Heemskerk J. W. Schmidt H. Hammer U. Heinz E. (1991). Biosynthesis and desaturation of prokaryotic galactolipids in leaves and isolated chloroplasts from spinach. Plant Physiol. 96, 144–152. 10.1104/pp.96.1.144, PMID: 16668143 PMC1080725

[ref11] Hicks G. R. Hironaka C. M. Dauvillee D. Funke R. P. D’Hulst C. Waffenschmidt S.. (2001). When simpler is better. Unicellular green algae for discovering new genes and functions in carbohydrate metabolism. Plant Physiol. 127, 1334–1338. 10.1104/pp.010821, PMID: 11743070 PMC1540159

[ref12] Hirashima T. Tajima N. Sato N. (2016). Draft genome sequences of four species of *Chlamydomonas* containing phosphatidylcholine. Genome Announc. 4, e01070–e01016. 10.1128/genomeA.01070-16, PMID: 27688324 PMC5043572

[ref13] Hu Q. Sommerfeld M. Jarvis E. Ghirardi M. Posewitz M. Seibert M.. (2008). Microalgal triacylglycerols as feedstocks for biofuel production: perspectives and advances. Plant J. 54, 621–639. 10.1111/j.1365-313X.2008.03492.x, PMID: 18476868

[ref14] Iwai M. Ikeda K. Shimojima M. Ohta H. (2014). Enhancement of extraplastidic oil synthesis in *Chlamydomonas reinhardtii* using a type-2 diacylglycerol acyltransferase with a phosphorus starvation-inducible promoter. Plant Biotechnol. J. 12, 808–819. 10.1111/pbi.12210, PMID: 24909748 PMC4160818

[ref15] James D. W. J. Lim E. Keller J. Plooy I. Ralston E. Dooner H. K. (1995). Directed tagging of the Arabidopsis *FATTY ACID ELONGATION1* (*FAE1*) gene with the maize transposon activator. Plant Cell 7, 309–319. 10.1105/tpc.7.3.309, PMID: 7734965 PMC160784

[ref16] Juergens M. T. Disbrow B. Shachar-Hill Y. (2016). The relationship of triacylglycerol and starch accumulation to carbon and energy flows during nutrient deprivation in *Chlamydomonas reinhardtii*. Plant Physiol. 171, 2445–2457. 10.1104/pp.16.00761, PMID: 27325664 PMC4972295

[ref17] Li X. Moellering E. R. Liu B. Johnny C. Fedewa M. Sears B. B.. (2012). A galactoglycerolipid lipase is required for triacylglycerol accumulation and survival following nitrogen deprivation in *Chlamydomonas reinhardtii*. Plant Cell 24, 4670–4686. 10.1105/tpc.112.105106, PMID: 23161887 PMC3531859

[ref18] Li-Beisson Y. Beisson F. Riekhof W. (2015). Metabolism of acyl-lipids in *Chlamydomonas reinhardtii*. Plant J. 82, 504–522. 10.1111/tpj.12787, PMID: 25660108

[ref19] Li-Beisson Y. Shorrosh B. Beisson F. Andersson M. Arondel V. Bates P.. (2013). “Acyl-lipid metabolism” in The Arabidopsis book. Vol. 11. Rockville, MD: American Society of Plant Biologists, e0161. 10.1199/tab.0161 PMC3563272 23505340

[ref20] Lohscheider J. N. Bártulos C. R. (2016). Plastoglobules in algae: a comprehensive comparative study of the presence of major structural and functional components in complex plastids. Mar. Genomics 28, 127–136. 10.1016/j.margen.2016.06.005, PMID: 27373732

[ref21] Lung S.-C. Weselake R. J. (2006). Diacylglycerol acyltransferase: a key mediator of plant triacylglycerol synthesis. Lipids 41, 1073–1088. 10.1007/s11745-006-5057-y, PMID: 17269553

[ref22] Menetrez M. Y. (2012). An overview of algae biofuel production and potential environmental impact. Environ. Sci. Technol. 46, 7073–7085. 10.1021/es300917r, PMID: 22681590

[ref23] Merchant S. A. Kropat J. Liu B. Shaw J. Warakanont J. (2012). TAG, You’re it! Chlamydomonas as a reference organism for understanding algal triacylglycerol accumulation. Curr. Opin. Biotechnol. 23, 352–363. 10.1016/j.copbio.2011.12.001, PMID: 22209109

[ref24] Millar A. A. Kunst L. (1997). Very-long-chain fatty acid biosynthesis is controlled through the expression and specificity of the condensing enzyme. Plant J. 12, 121–131. 10.1046/j.1365-313X.1997.12010121.x, PMID: 9263455

[ref25] Misra N. Panda P. K. Parida B. K. Mishra B. K. (2012). Phylogenomic study of lipid genes involved in microalgal biofuel production—candidate gene mining and metabolic pathway analyses. Evol. Bioinform. 8, 545–564. 10.4137/EBO.S10159, PMID: 23032611 PMC3460774

[ref26] Moellering E. R. Benning C. (2010). RNA interference silencing of a major lipid droplet protein affects lipid droplet size in *Chlamydomonas reinhardtii*. Eukaryot. Cell 9, 97–106. 10.1128/EC.00203-09, PMID: 19915074 PMC2805299

[ref27] Mori N. Moriyama T. Sato N. (2019). Uncommon properties of lipid biosynthesis of isolated plastids in the unicellular red alga *Cyanidioschyzon merolae*. FEBS Open Bio. 9, 114–128. 10.1002/2211-5463.12551, PMID: 30652079 PMC6325583

[ref28] Moriyama T. Toyoshima M. Saito M. Wada H. Sato N. (2018). Revisiting the algal “chloroplast lipid droplet”: the absence of an entity that is unlikely to exist. Plant Physiol. 176, 1519–1530. 10.1104/pp.17.01512, PMID: 29061905 PMC5813570

[ref29] Moseley K. R. Thompson G. A. (1980). Lipid composition and metabolism of volvox carteri. Plant Physiol. 65, 260–265. 10.1104/pp.65.2.260, PMID: 16661171 PMC440308

[ref30] Msanne J. Xu D. Konda A. R. Casas-Mollano J. A. Awada T. Cahoon E. B.. (2012). Metabolic and gene expression changes triggered by nitrogen deprivation in the photoautotrophically grown microalgae *Chlamydomonas reinhardtii* and *Coccomyxa* sp. C-169. Phytochemistry 75, 50–59. 10.1016/j.phytochem.2011.12.007, PMID: 22226037

[ref31] Riekhof W. R. Sears B. B. Benning C. (2005). Annotation of genes involved in glycerolipid biosynthesis in *Chlamydomonas reinhardtii*: discovery of the betaine lipid synthase BTA1Cr. Eukaryot. Cell 4, 242–252. 10.1128/EC.4.2.242-252.2005, PMID: 15701786 PMC549322

[ref32] Sakurai K. Moriyama T. Sato N. (2014). Detailed identification of fatty acid isomers sheds light on the probable precursors of triacylglycerol accumulation in photoautotrophically grown *Chlamydomonas reinhardtii*. Eukaryot. Cell 13, 256–266. 10.1128/EC.00280-13, PMID: 24337111 PMC3910977

[ref33] Sato N. (1991). Lipids in Cryptomonas CR-1. II. Biosynthesis of betaine lipids and galactolipids. Plant Cell Physiol. 32, 845–851. 10.1093/oxfordjournals.pcp.a078152

[ref34] Sato N. (2015). Is monoglucosyl diacylglycerol a precursor to monogalactosyl diacylglycerol in all cyanobacteria? Plant Cell Physiol. 56, 1890–1899. 10.1093/pcp/pcv116 26276824

[ref35] Sato N. Moriyama T. (2007). Genomic and biochemical analysis of lipid biosynthesis in the unicellular rhodophyte *Cyanidioschyzon merolae*: lack of plastidic desaturation pathway results in mixed pathway of galactolipid synthesis. Eukaryot. Cell 6, 1006–1017. 10.1128/EC.00393-06, PMID: 17416897 PMC1951526

[ref36] Sato N. Moriyama T. Mori N. Toyoshima M. (2017). Lipid metabolism and potentials of biofuel and high added-value oil production in red algae. World J. Microbiol. Biotechnol. 33:74. 10.1007/s11274-017-2236-3. 28303457

[ref37] Sato N. Murata N. (1982). Lipid biosynthesis in the blue-green alga, *Anabaena variabilis* I. lipid classes. Biochim. Biophys. Acta 710, 271–278. 10.1016/0005-2760(82)90109-6

[ref38] Sato N. Obayashi T. (2021). “Lipid pathway databases with a focus on algae” in Methods in molecular biology: Plant lipids. eds. Bartels D. Dörmann P. (Springer Science) (in press). 10.1007/978-1-0716-1362-7_26 34047993

[ref39] Sato N. Okazaki Y. Saito K. (2016). Isotopic combinatomer analysis provides *in vivo* evidence of the direct epimerization of monoglucosyl diacylglycerol in cyanobacteria. Biochemistry 55, 5689–5701. 10.1021/acs.biochem.6b00769, PMID: 27653026

[ref40] Siaut M. Cuiné S. Cagnon C. Fessler B. Nguyen M. Carrier P.. (2011). Oil accumulation in the model green alga *Chlamydomonas reinhardtii*: characterization, variability between common laboratory strains and relationship with starch reserves. BMC Biotechnol. 11:7. 10.1186/1472-6750-11-7, PMID: 21255402 PMC3036615

[ref41] Singh H. Shukla M. R. Chary K. V. R. Rao B. J. (2014). Acetate and bicarbonate assimilation and metabolite formation in *Chlamydomonas reinhardtii*: a ^13^C-NMR study. PLoS One 9:e106457. 10.1371/journal.pone.0106457, PMID: 25207648 PMC4160175

[ref42] Smith R. T. Gilmour D. J. (2018). The influence of exogenous organic carbon assimilation and photoperiod on the carbon and lipid metabolism of *Chlamuydomonas reinhardtii*. Algal Res. 31, 122–137. 10.1016/j.algal.2018.01.020

[ref43] Tornabene T. G. Holzer G. Lien S. Burris N. (1983). Lipid composition of the nitrogen starved green alga *Neochloris oleoabundans*. Enzym. Microb. Technol. 5, 435–440. 10.1016/0141-0229(83)90026-1

[ref44] Toyoshima M. Sato N. (2015). High-level accumulation of triacylglycerol and starch in photoautotrophically grown *Chlamydomonas debaryana* NIES-2212. Plant Cell Physiol. 56, 2447–2456. 10.1093/pcp/pcv163, PMID: 26542110

[ref45] Toyoshima M. Sato N. (2018). Optimization of triacylglycerol and starch production in *Chlamydomonas debaryana* NIES-2212 with regard to light intensity and CO_2_ concentration. Microbiology 164, 359–368. 10.1099/mic.0.000603, PMID: 29458672

[ref46] Van den Koornhuyse N. Libessart N. Delrue B. Zabawinski C. Decq A. Iglesias A.. (1996). Control of starch composition and structure through substrate supply in the monocellular alga *Chlamydomonas reinhardtii*. J. Biol. Chem. 271, 16281–16287. 10.1074/jbc.271.27.16281, PMID: 8663144

[ref47] Watanabe A. (1960). List of algal strains in collection at the institute of applied microbiology, University of Tokyo. J. Gen. Appl. Microbiol. 6, 283–292. 10.2323/jgam.6.283

[ref48] Williams J. P. Watson G. R. Khan M. U. Leung S. (1975). Galactolipid synthesis in *Vicia faba* leaves: I. Galactose, glycerol, and fatty acid labeling after CO_2_ feeding. Plant Physiol. 55, 1038–1042. 10.1104/pp.55.6.1038, PMID: 16659205 PMC541761

[ref49] Yoshitomi T. Kaminaga S. Sato N. Toyoshima M. Moriyama T. Yoshimoto K. (2020). Formation of spherical palmelloid colony with enhanced lipid accumulation by gel encapsulation of *Chlamydomonas debaryana* NIES-2212. Plant Cell Physiol. 61, 158–168. 10.1093/pcp/pcz188, PMID: 31589321

[ref50] Yumoto K. Kasai F. Kawachi M. (2013). Taxonomic re-examination of *Chlamydomonas* strains maintained in the NIES-collection. Microbiol. Cult. Coll. 2, 1–12.

[ref51] Yusuf C. (2007). Biodiesel from microalgae. Biotechnol. Adv. 25, 294–306. 10.1016/j.biotechadv.2007.02.001. 17350212

[ref52] Zhang B. Wang L. Hasan R. Shahbazi A. (2014). Characterization of a native algae species *Chlamydonmonas debaryana*: strain selection, bioremediation ability, and lipid characterization. Bioresources 9, 6130–6140. 10.15376/biores.9.4.6130-6140

