# Supplementary material for: Dynamism of Metabolic Carbon Flow of Starch and Lipids in Chlamydomonas debaryana
Source: Front Plant Sci. 2021 Mar 30;12:646498. doi: 10.3389/fpls.2021.646498 (PMC8047662; doi:10.3389/fpls.2021.646498)
Supplement: Supplementary file 1 [file Data_Sheet_1.pdf]

Supplementary Material

Appendix: First-order kinetics of isotopically labeled metabolites

In the *in vivo* labeling experiments dependent on photosynthesis of a natural carbon source $^{13}CO_2$ (fed as $NaH^{13}CO_3$), any synthesized metabolite will consist of a labeled population and an unlabeled one. We consider a metabolite A, which is subject to the following metabolism (Fig. A1).

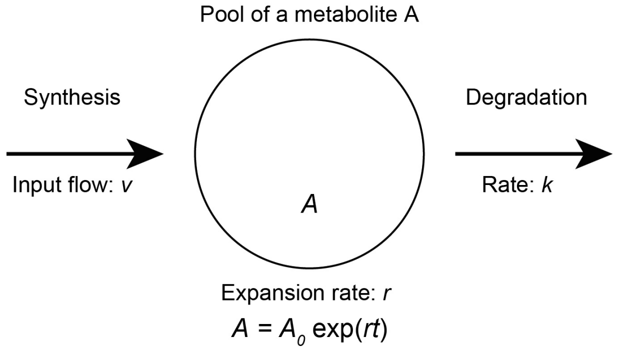

Fig. A1. Model of metabolic flow

The pool size *A* will increase with the rate of expansion *r*, which is equal to the growth rate *g* in the steady-state:

$$A = A_0 \exp{(rt)} \text{ --- (1)}$$

The actual growth rate of *Chlamydomonas debaryana* cells *g* was estimated as 0.0257 h^{-1} in the exponential growth phase, which corresponds to a doubling time of 27 h. The rate *r* was expected to be in a similar order to *g*. The expansion of the pool size of A is a balance of its synthesis *v* and its degradation with the first-order kinetic constant *k*:

$$\frac{dA}{dt} = v - kA \text{ --- (2)}$$

The rate of synthesis *v* is the sum of the increase in pool size and the degradation:

$$v = (k + r)A \text{ --- (3)}$$

In the labeling phase, newly synthesized A consists entirely of a labeled population, whereas the degradation occurs for both labeled and unlabeled A. The rate of increase in the labeled population A* is described by the following equation:

$$\frac{dA^*}{dt} = (k + r)A - kA^* \text{ --- (4)}$$

The solution is given by the following equation:

$$A^* = A - A_0 \exp(-kt) \text{ --- (5)}$$

The proportion x of labeled population within the total pool of A is expressed in the following way:

$$x = \frac{A^*}{A} = 1 - \exp[-(k + r)t] = 1 - \exp(-k't) \text{ --- (6)}$$

where $k' = k + r$. In the chase experiments, the metabolite A contains a labeled population A^*, which decreases with time, whereas a newly synthesized population consists entirely of unlabeled one. The proportion x of labeled population will change according to the following equation:

$$x = x_0 \exp(-k't) \text{ --- (7)}$$

where x_0 is the initial level of x at the start of chase.

The k' value can be obtained by either the labeling experiments according to the equation (6), or the chase experiments according to the equation (7). In the actual analysis, the chase kinetics consists of rapid and slow processes, which are estimated by the non-linear least square method with the 'fit' function of GNUPLOT version 5. The proportion of labeled population x will then be expressed by the following equation:

$$x = 1 - [a_1 \exp(-k'_1 t) + a_2 \exp(-k'_2 t)] \text{ --- (8)}$$

where a_1 and a_2 are the size of the two pools (rapid and slow), respectively, and k'_1 and k'_2 are the rate constant of degradation of the respective pools. The single value k' obtained by the labeling rate was not always consistent with the two values k'_1 and k'_2 for unknown reasons.

Note that the above description is based on perfectly labeled and unlabeled populations. However, the actual experiments detected poorly labeled and intermediately labeled populations. Nevertheless, the consideration as described above is valid in these cases, because each differently labeled population can be treated independently as A* within the total pool A.
